# APPsα rescues CDK5 and GSK3β dysregulation and restores normal spine density in Tau transgenic mice

**DOI:** 10.3389/fncel.2023.1106176

**Published:** 2023-01-26

**Authors:** Danny Baltissen, Charlotte S. Bold, Lena Rehra, Marija Banićević, Justus Fricke, Jennifer Just, Susann Ludewig, Christian J. Buchholz, Martin Korte, Ulrike C. Müller

**Affiliations:** ^1^Department of Functional Genomics, Institute of Pharmacy and Molecular Biotechnology, Heidelberg University, Heidelberg, Germany; ^2^Department of Cellular Neurobiology, Zoological Institute, Technical University of Braunschweig, Braunschweig, Germany; ^3^Department of Molecular Biotechnology and Gene Therapy, Paul-Ehrlich-Institut, Langen, Germany; ^4^Helmholtz Centre for Infection Research, Neuroinflammation and Neurodegeneration Group, Braunschweig, Germany

**Keywords:** Alzheimer’s disease, Tau, CDK5, GSK3β, APPsα, THY-Tau22, Tau hyperphosphorylation, spines

## Abstract

The Tau protein can be phosphorylated by numerous kinases. In Alzheimer’s disease (AD) hyperphosphorylated Tau species accumulate as neurofibrillary tangles that constitute a major hallmark of AD. AD is further characterized by extracellular Aβ plaques, derived from the β-amyloid precursor protein APP. Whereas Aβ is produced by amyloidogenic APP processing, APP processing along the competing non-amyloidogenic pathway results in the secretion of neurotrophic and synaptotrophic APPsα. Recently, we demonstrated that APPsα has therapeutic effects in transgenic AD model mice and rescues Aβ-dependent impairments. Here, we examined the potential of APPsα to regulate two major Tau kinases, GSK3β and CDK5 in THY-Tau22 mice, a widely used mouse model of tauopathy. Immunohistochemistry revealed a dramatic increase in pathologically phosphorylated (AT8 and AT180) or misfolded Tau species (MC1) in the hippocampus of THY-Tau22 mice between 3 and 12 months of age. Using a highly sensitive radioactive kinase assay with recombinant human Tau as a substrate and immunoblotting, we demonstrate an increase in GSK3β and CDK5 activity in the hippocampus of THY-Tau22 mice. Interestingly, AAV-mediated intracranial expression of APPsα in THY-Tau22 mice efficiently restored normal GSK3β and CDK5 activity. Western blot analysis revealed upregulation of the CDK5 regulatory proteins p35 and p25, indicating CDK5 hyperactivation in THY-Tau22 mice. Strikingly, AAV-APPsα rescued p25 upregulation to wild-type levels even at stages of advanced Tau pathology. Sarkosyl fractionation used to study the abundance of soluble and insoluble phospho-Tau species revealed increased soluble AT8-Tau and decreased insoluble AT100-Tau species upon AAV-APPsα injection. Moreover, AAV-APPsα reduced misfolded (MC1) Tau species, particularly in somatodendritic compartments of CA1 pyramidal neurons. Finally, we show that AAV-APPsα upregulated PSD95 expression and rescued deficits in spine density of THY-Tau22 mice. Together our findings suggest that APPsα holds therapeutic potential to mitigate Tau-induced pathology.

## 1. Introduction

Alzheimer’s disease (AD) is histopathologically characterized by extracellular Aβ plaques and the intracellular accumulation and aggregation of Tau species, the latter also constituting key hallmarks of primary tauopathies [reviewed in Iqbal et al. (2010)]. Tau was originally identified as a microtubule-associated protein that can enhance microtubule assembly and stability (Weingarten et al., 1975; Cleveland et al., 1977). During recent years it has become clear that Tau can exert a multitude of physiological functions not only in the axon, where it binds to microtubules and regulates axonal transport, but also in dendrites, the nucleus and at the synapse (Wang and Mandelkow, 2016; Brandt et al., 2020; Mueller et al., 2021). Monomeric Tau is a natively unfolded protein that is particularly rich in proline and lysine residues and contains 85 potential phosphorylation sites. Under pathological conditions including AD, Tau becomes increasingly hyperphosphorylated, resulting from a decrease in the activity of phosphatases and/or an increase in the activity of kinases, which leads to the detachment of Tau from microtubules. This promotes its mislocalization to the somatodendritic compartment and a transition from soluble to insoluble oligomers that fibrillize and are finally deposited as NFTs (Fitzpatrick et al., 2017; Wegmann et al., 2018). Phosphorylation of Tau is under tight control of various protein kinases (Li and Götz, 2017), including glycogen synthase kinase 3β (GSK3β) and cyclin dependent kinase 5 (CDK5), that are considered as major pathologic Tau kinases (Baumann et al., 1993; Paudel et al., 1993; Kremer et al., 2011; Lauretti et al., 2020). GSK3β is a widely expressed multifunctional serine/threonine kinase that phosphorylates Tau predominantly at Ser^199^, Ser^96^, and Ser^413^ in tauopathies (Avila et al., 2010). In addition, increased GSK3β activity was reported to induce Aβ formation and has also been implicated in neuroinflammation and neuronal death (Cai et al., 2012).

CDK5 is a proline directed serine/threonine protein kinase involved in numerous physiological and pathological functions within the CNS (Pao and Tsai, 2021). CDK5 is an atypical cyclin-dependent kinase, that requires association with regulatory proteins p35 or p39 for activation. Importantly, neurotoxic insults trigger the activation of calpain, that cleaves p35 to generate p25, in a Ca^2+^-dependent manner. p25 in turn leads to dysregulation and hyperactivation of CDK5, including cellular mislocalization that redirects CDK5 to additional substrates under pathological conditions [reviewed in Kimura et al. (2014) and Pao and Tsai (2021)]. Tau can be phosphorylated by CDK5 at multiple sites (Lau et al., 2002) including Ser^202^, Thr^205^, Ser^396^, and Ser^404^ as major sites in AD patients (Shukla et al., 2012). Together, GSK3β and CDK5 constitute important therapeutic targets for AD, that have also prompted the development of respective kinase inhibitors (Li and Götz, 2017; Yu et al., 2021), although none has as yet shown clinical benefit.

Aβ is generated by sequential cleavage of the amyloid precursor protein (APP) by β- and γ-secretase [Lichtenthaler et al., 2011, for review see Lichtenthaler et al. (2022)]. In the competing and physiologically predominant non-amyloidogenic pathway, APP is cleaved by α-secretase (Lammich et al., 1999) which precludes the formation of Aβ and liberates the neuroprotective ectodomain APPsα. Shifting APP processing toward non-amyloidogenic processing has therefore been suggested as a therapeutic strategy for AD (Mockett et al., 2017; Müller et al., 2017). Indeed, accumulating evidence indicates that APPsα has physiological properties that make it an attractive therapeutic target. Previous studies from us and others indicated that APPsα has neurotrophic and neuroprotective effects *in vitro* and *in vivo* (Müller et al., 2017), most notably synaptogenic, LTP facilitating and memory enhancing properties (Meziane et al., 1998; Ring et al., 2007; Taylor et al., 2008; Weyer et al., 2011, 2014; Tyan et al., 2012; Milosch et al., 2014; Hick et al., 2015; Zou et al., 2016; Xiong et al., 2017; Richter et al., 2018; Mockett et al., 2019). In addition, APPsα had protective effects in models of acute neuronal injury including hypoxia-ischemia (Smith-Swintosky et al., 1994; Hefter et al., 2016) and traumatic head injury (Corrigan et al., 2012; Plummer et al., 2016), that has been associated with an increased risk to develop dementia and AD (Li et al., 2017).

Recently, we showed that APPsα has therapeutic potential in a transgenic mouse model with Aβ plaque pathology (APP/PS1ΔE9 mice). APPsα expression in the hippocampus of these mice rescued deficits in synaptic plasticity, spine density and spatial memory (Fol et al., 2016). These results raised the question of whether the beneficial *in vivo* effects of APPsα may also be exploited for Aβ-independent impairments, in particular Tau-induced pathology (Bold et al., 2022). In this regard, previous studies had indicated that APPsα overexpression in neuroblastoma cells can inhibit GSK3β (Deng et al., 2015) and that incubation of primary neurons with recombinant APPsα (recAPPsα) resulted in downregulation of CDK5 expression and activity (Hartl et al., 2013) *in vitro*.

Here, we used the THY-Tau22 mouse line (Schindowski et al., 2006) as an established model of tauopathy to examine whether APPsα may modulate GSK3β and CDK5 kinase activity *in vivo* and ameliorate Tau-induced pathology and synaptic deficits *in vivo.*

## 2. Results

### 2.1. Progressive Tau pathology in the hippocampus of THY-Tau22 mice

**FIGURE 1:**
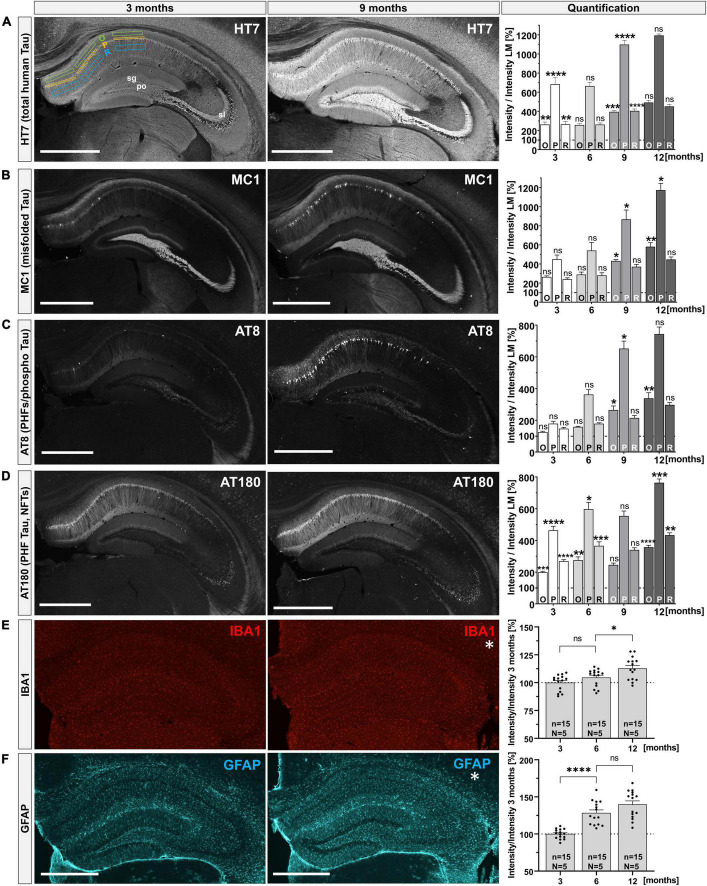
Progressive Tau pathology in the hippocampus of THY-Tau22 mice. **(A–D)** Distinct antibodies were used to detect total human Tau (HT7), misfolded Tau (MC1), PHFs and phospho-Tau (AT8 and AT180) at 3, 6, 9, and 12 months of age in hippocampi of THY-Tau22 mice. Exemplary images are shown for 3 (left) and 9 months (middle). Quantification of immunoreactivity over time (right): immunoreactivity for each antibody was measured in three layers of the CA1 region (stratum oriens = O; stratum pyramidale = P; and stratum radiatum = R), by placing rectangles into each area and measuring mean fluorescence intensities (MFI), as shown for HT7 staining at 3 months [**(A)**, left]. Staining intensity in the CA1 layers was quantified relative to staining intensity of wild type controls (set as 100%). For the first time point (3 months of age), significant differences are indicated relative to wild type controls. For subsequent time points (6, 9, and 12 months of age) differences in immunoreactivity in THY-Tau22 sections were calculated relative to the proceeding time point to asses age-dependent differences in each of the layers analyzed. **(A)** At 3 months HT7^+^ immunoreactivity was present in all subfields (CA1, CA3, and DG) of the hippocampus (left). At 9 months signal intensity increased in all subfields (middle), leading to a significant increase in the mean pixel intensities for all subfields over time (right). **(B)** At 3 months misfolded Tau species (MC1^+^) were mainly detected in the polymorph layer (po) of the DG, the stratum lucidum (sl) and the distal stratum pyramidale (P) of CA1 (left). At 9 months the number of MC1^+^ cells located in CA1 region was increased (middle), resulting in increased MFIs in O and P (right). **(C)** At 3 months only weak AT8^+^ immunoreactivity was detectable, with few scattered positive cells in the CA1 region (left). At 9 months AT8^+^ immunoreactivity was detectable in dentate granule cells and was dramatically increased in CA1 pyramidal cells (middle), resulting in increased MFIs in O and P (right). **(D)** At 3 months of age, AT180^+^ immunoreactivity was prominent in all hippocampal subfields (left) and was even more enhanced at later time-points (9 months, middle). Quantification indicated a progressive increase from 3 to 12 months of age in all layers (right). **(E)** Microglia (IBA1) were present in a regularly distributed pattern all over the hippocampus with no obvious changes in morphology or distribution over time (left and middle), despite a slight increase in MFI at 12 months compared to 6 months of age (right). Note that no analysis was performed for IBA and GFAP at 9 months of age. **(F)** With exception for the stratum pyramidale and stratum granulosum (sg), also astroglia (GFAP) were regularly distributed over the whole hippocampus. From 3 to 6 months of age there was a significant increase in GFAP immunoreactivity. Data are depicted as mean ± SEM. MFIs in sublayers were analyzed using one-way ANOVA with Bonferroni’s *post hoc* test: *p ≤ 0.05, **p ≤ 0.01, ***p ≤ 0.001, ****p ≤ 0.0001. All images were captured with the same laser intensity, avoiding overexposure. *N* = 3–5 animals per genotype and age, *n* = 6–15 analyzed areas; images depict maximum intensity projections taken from 40 μm coronal sections, scale bars: 500 μm.

Previous studies indicated that THY-Tau22 mice develop Tau pathology from the age of 3 months onwards, including hyperphosphorylated and misfolded Tau species that were associated with changes in synaptic plasticity and cognitive impairments, (Schindowski et al., 2006; van der Jeugd et al., 2011, 2013; Ahmed et al., 2015; Vautheny et al., 2021). Here, we started by performing a systematic analysis of the temporal time course of Tau pathology to cover low (3 months), intermediate (6 months) and severe stages of pathology (9 and 12 months). We focused on the hippocampus due to its key role in learning and memory, synaptic plasticity, and as a brain region affected very early during AD pathogenesis. Hippocampal section of THY-Tau22 mice were stained with a panel of different antibodies to visualize total human Tau (HT7), misfolded Tau (MC1) and abnormally hyperphosphorylated Tau (AT8 and AT180). Immunoreactivity was quantified in comparison to wild-type littermate controls by measuring the mean fluorescence intensity (MFI) in the CA1 subfield of the hippocampus, a region that is particularly vulnerable to neurodegeneration in AD (Padurariu et al., 2012). To analyze the somatodendritic localization of Tau pathology in more detail we quantified immunoreactivity (as measured by MFI) in several hippocampal layers including the stratum oriens (O) containing the basal dendrites of CA1 neurons, the stratum pyramidale (P) containing cell bodies, and the stratum radiatum (R), containing apical dendrites of pyramidal neurons (Benavides-Piccione et al., 2020). Total hTau (HT7 staining), was detected in all subregions of the hippocampus (CA1, CA3, and DG) from 3 months onwards and strongly increased over time (Figure 1A, O: 3 months 262.25 ± 26.81% vs. 12 months 492.58 ± 21.92%, ^****^p < 0.0001; P: 3 months 683.28 ± 69.61% vs. 12 months 1193.17 ± 16.39%, ^***^p = 0.0001; R: 3 months 263.71 ± 26.10% vs. 12 months 451.62 ± 21.65%, ^***^p = 0.0003). Abnormal conformational Tau species were detected using the MC1 antibody, directed against a conformational epitope formed by aa^7–9^ and aa^326–330^ that get into close proximity upon misfolding of Tau (Jicha et al., 1997). MC1 immunoreactivity was most prominent in the mossy fibers and was also detectable in pyramidal cells in distal CA1 at 3 months of age (Figure 1B, left). With progressing age (at 9 and 12 months), the number of MC1-positive cells in CA1 increased particularly in proximal CA1 regions, leading to a significant increase in mean fluorescence intensity in stratum pyramidale and stratum oriens (Figure 1B, middle and right). Using the AT8 antibody we detected hTau phosphorylated at Ser^202^, Thr^205^, and Ser^208^. At 3 months of age, only very weak AT8 immunoreactivity was observed with very few AT8^+^ neurons in distal CA1 (Figure 1C, left). At later time points (at 9 and 12 months), AT8 immunoreactivity was prominently detected in many neuronal cell bodies along the whole proximal to distal CA1 region and within stratum oriens (Figure 1C, middle). Paired helical filament hTau, phosphorylated at Thr^231^, was detected using the AT180 antibody. Already at 3 months of age, we detected intense AT180 staining in distal and proximal CA1 regions within all hippocampal layers including stratum lacunosum moleculare and also in the CA3 subregion (Figure 1D, left). With progressing age, AT180 immunoreactivity further increased both in dendritic areas (O and R), as well as in somata (P) (Figure 1D, middle and right). No specific staining was observed in littermate control mice with any of the used anti-Tau antibodies (Supplementary Figure 1). In addition, we also assessed potential gliosis using IBA1 as a marker for activated microglia (Figure 1E) and GFAP to detect reactive astrocytes (GFAP, Figure 1F). We didn’t observe any differences in the distribution or gross morphology of microglia with increasing age albeit mean IBA^+^ fluorescence intensity slightly increased from 6 to 12 months (Figure 1E; right, 6 months 104.60 ± 2.02% vs. 12 months 112.73 ± 2.61%, *p = 0.032). In contrast, we observed a moderate astrogliosis as indicated by increased GFAP^+^ immunoreactivity with age (Figure 1F; right, 3 months 100.00 ± 1.68% vs. 12 months 139.82 ± 4.84%, ^****^p < 0.0001). Together, we show that THY-Tau22 mice express hTau in all hippocampal subfields and exhibit an age-dependent progressive increase of Tau pathology characterized by Tau hyperphosphorylation, Tau misfolding and a pronounced somatodendritic localization of pathological Tau species. This age-related increase in Tau pathology is also accompanied by mild astrogliosis.

### 2.2. Assessment of CDK5 and GSK3β kinase activity in THY-Tau22 mice using a radioactive kinase assay

**FIGURE 2:**
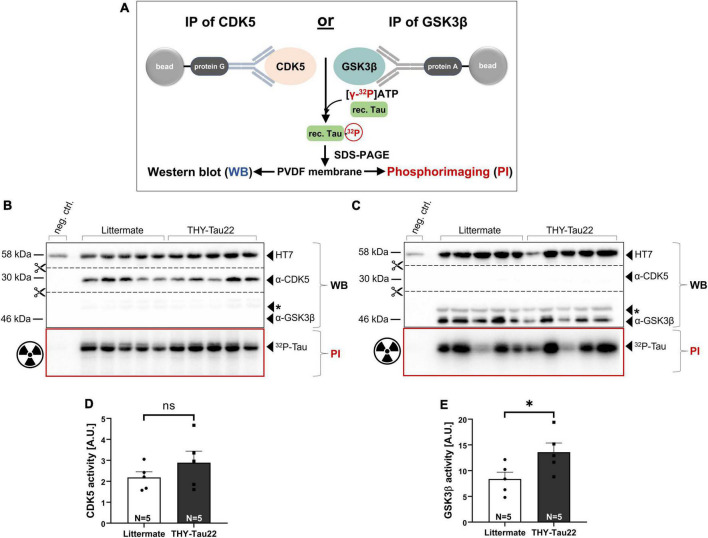
Assessment of kinase activity in THY-Tau22 mice using a radioactive kinase assay. **(A)** Schematic overview of assay format. GSK3β or CDK5-activator complexes are immunoprecipitated from hippocampal tissue homogenates using protein G/A coupled beads. Immunoprecipitated kinases were incubated with [γ−^32^P]-ATP and recombinant Tau, leading to radioactive labeling. For further analysis samples were separated by SDS-PAGE, transferred to a PVDF membrane and visualized using Western blot and phosphorimaging. **(B,C)** Western blot (WB) and phosphorimaging (PI) analysis after immunoprecipitation of **(B)** CDK5 or **(C)** GSK3β from littermate controls and THY-Tau22 mice at 12 months of age. 650 μg of total hippocampal lysate was used as input for each IP when employing the α-CDK5 antibody **(B)**. The remaining lysate (amounting to about 300–600 μg) was used for GSK3β-IP **(C)**. As total protein yield varied between the hippocampal samples the second IP was performed with more variable input, as reflected in the more variable GSK3β WB signal. To account for input variability signal intensity of ^32^P-recTau (PI) was normalized to total amount of immunoprecipitated CDK5 (α-CDK5 WB signal intensity) **(B)** or to total amount of immunoprecipitated GSK3β (α-GSK3β WB signal intensity) **(C)**, respectively. Kinase activity was plotted as arbitrary units (A.U.). Note the absence of GSK3β after immunoprecipitation with an CDK5-specific antibody **(B)** and the absence of CDK5 after immunoprecipitation with an GSK3β-specific antibody **(C)**. The asterisk * indicates an unspecific band detected after α-GSK3β staining. **(D)** CDK5 activity increased in THY-Tau22 mice without reaching significance (Littermate vs. THY-Tau22, *p* = 0.29, ns). **(E)** GSK3β activity was significantly increased in THY-Tau22 mice compared to littermate controls (Littermate vs. THY-Tau22, *p = 0.04). Data are depicted as mean ± SEM; N, number of animals; age, 12 months; data were analyzed using a two-tailed Student’s *t*-test.

Transgenic THY-Tau22 mice overexpress the human 1N4R Tau isoform mutated at sites P301S and G272V, a Tau mutation which has previously been shown to promote the hyperphosphorylation of Tau by several protein kinases (Del Alonso et al., 2004). Here, we asked the question of whether Tau hyperphosphorylation, as evidenced by our immunohistochemical study (see Figures 1A–D), is associated with and may be due to aberrant kinase activity in the hippocampus of THY-Tau22 mice (Del Alonso et al., 2004). We focused on CDK5 and GSK3β, two kinases previously implicated in AD (Engmann and Giese, 2009). To this end, GSK3β or CDK5-activator complexes were immunoprecipitated from hippocampal lysates of THY-Tau22 and LM control mice (at 12 months of age), and their activity was determined using a highly sensitive radioactive kinase assay with recombinant 1N4R Tau as the substrate (see Figure 2A), the human Tau isoform overexpressed in THY-Tau22 mice. Briefly, incubation of immunoprecipitated native kinases with [γ-^32^P]-ATP leads to the radioactive labeling of recTau, as evidenced by phosphorimaging after SDS-PAGE. Subsequent Western blot analysis of the same membrane allows to detect the total amounts of kinase precipitated. Kinase activity is measured as the ratio of ^32^P-recTau (PI, phosphorimaging) normalized to the total amounts of kinase precipitated (WB signal). Figure 2 shows representative Western blots and phosphorimaging micrographs after CDK5 immunoprecipitation (Figure 2B), or after GSK3β pull-down, respectively (Figure 2C). Upon pull-down with an antibody directed against CDK5, we failed to detect any GSK3β-specific signal and similarly, could not detect any CDK5 signal after immunoprecipitation with an antibody directed against GSK3β. Together, this indicates that the activity of both kinases can be measured with high specificity in brain lysates. Quantification of kinase activity in hippocampal extracts of THY-Tau22 mice, revealed an increase in CDK5 activity that did, however, not reach significance (Figure 2D; Littermate 2.18 ± 0.27 A.U. vs. THY-Tau22: 2.89 ± 0.55 A.U., *p* = 0.29, ns). Compared to littermate controls, THY-Tau22 mice showed a significant about 1.6-fold increase of GSK3β activity (Figure 2E; Littermate 8.38 ± 1.32 A.U. vs. THY-Tau22 13.59 ± 1.78 A.U., *p = 0.047), that likely contributes to hTau hyperphosphorylation in THY-Tau22 hippocampus.

### 2.3. APPsα restores normal GSK3β activity and modulates the Akt/GSK3β pathway in THY-Tau22 mice

**FIGURE 3:**
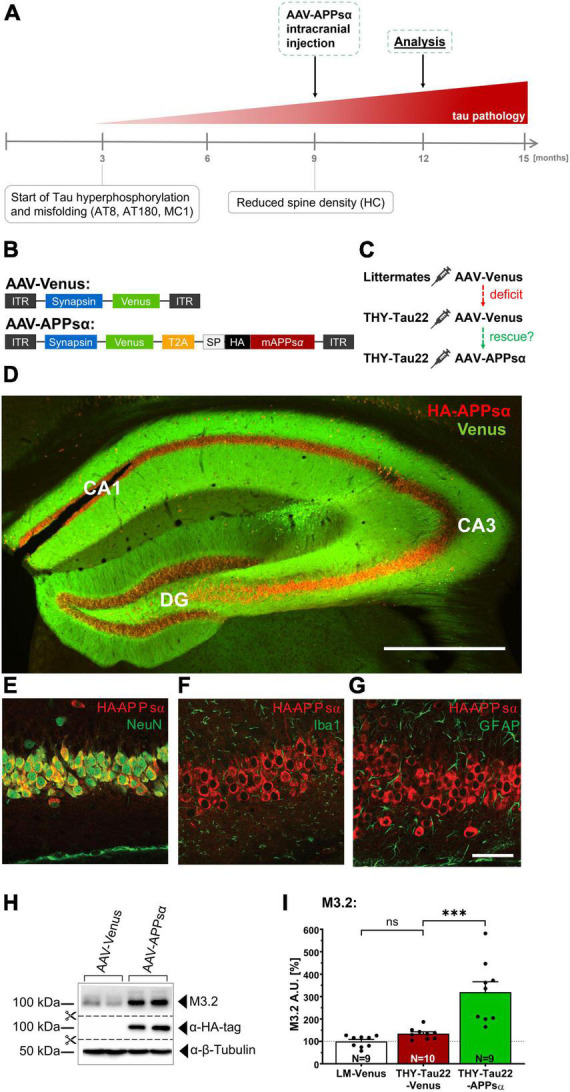
Efficient AAV-mediated expression of APPsα in THY-Tau22 hippocampus. **(A)** Scheme of study outline. THY-Tau22 animals were intracranially injected at 9 months of age, when Tau pathology is well established (curative approach) and mice exhibit reduced spine density. Age of analysis: 12 months. **(B)** Schematic representation of AAV9 constructs enabling the neuron-specific expression of Venus (control) and HA-tagged murine APPsα, under control of the synapsin promotor. Bicistronic AAV-APPsα was used to co-express Venus and APPsα. ITR, inverted terminal repeat; T2A, Thosea asigna virus 2A site; HA, influenza hemagglutinin tag. Note that Venus carries a membrane anchor. **(C)** Three experimental groups were treated. Wild type littermate controls received AAV-Venus. THY-Tau22 were either injected with AAV-Venus, or with AAV-APPsα. **(D)** Representative coronal brain slice of a THY-Tau22-APPsα injected mouse. HA-APPsα expression is detected throughout the hippocampus, evidenced by anti-HA-tag staining and Venus fluorescence. Scale bar: 500 μm. **(E–G)** The expression of AAV-APPsα is neuron-specific and co-localized with the neuronal biomarker NeuN **(E)**. **(F)** No co-staining of HA-APPsα was detected with IBA1 (microglia) or with GFAP (astrocytes) **(G)**. Scale bar: 100 μm. **(H)** Western blot analysis of hippocampal lysates of AAV-Venus or AAV-APPsα injected THY-Tau22 mice. The M3.2 antibody detects the vector-derived APPsα in addition to endogenous APP species. β-Tubulin is depicted as a qualitative loading control. Note that for quantification of M3.2 immunoreactivity a normalization was performed against total protein level per lane (stain-free method, Bio-Rad). **(I)** Quantification of the M3.2 immunoreactivity revealed a 3.2-fold increase in AAV-APPsα injected mice (THY-Tau22-Venus vs. THY-Tau22-APPsα, ^***^p = 0.0002). Data are depicted as mean ± SEM; N, number of animals; age, 12 months; data were analyzed using one-way ANOVA with Tukey *post hoc* test: ^***^p ≤ 0.001.

Previous *in vitro* studies had indicated that neurotrophic APPsα may regulate CDK5 and GSK3β (Hartl et al., 2013; Deng et al., 2015). Given the increase of CDK5 and GSK3β activity that we identified in the hippocampus of THY-Tau22 mice, we next aimed to test the potential of APPsα to normalize kinase activity. To this end, we used an AAV9-based vector to express APPsα in the dorsal hippocampus of THY-Tau22 mice *in vivo*. Previously, we had shown that AAV vector encoded APPsα is transported within the secretory pathway to the cell surface, resulting in efficient secretion of HA-APPsα into the extracellular space (Fol et al., 2016; Richter et al., 2018). Here, we performed stereotactic injections of a bicistronic viral vector (AAV-APPsα, Figure 3B), encoding the HA-tagged murine APPsα and fluorescent Venus, allowing to track transduced cells. A T2A site was used to fuse both expression cassettes, that were under control of the neuron-specific synapsin promotor (Figure 3B). Monocistronic AAV-Venus, coding only for Venus, served as a control (Figure 3B). In both vectors Venus carried a C-terminal farnesylation signal for membrane anchoring. To assess the therapeutic potential of APPsα we injected mice at 9 months of age, a stage when THY-Tau22 mice show severe Tau pathology and increased kinase activity [see Figure 3A and Schindowski et al. (2006)]. Transgenic THY-Tau22 mice received either AAV-APPsα or AAV-Venus. Wild type littermates served as a control group and received only AAV-Venus (Figure 3C). Viral vectors were bilaterally injected into the DG (first injection spot) and the stratum lacunosum-moleculare (second injection spot) of the dorsal hippocampus, similarly to our previous studies in Aβ overexpressing mice (Fol et al., 2016; Richter et al., 2018). Mice were sacrificed 3 months post injection at 12 months of age. First, we assessed expression of Venus and HA-APPsα by immunohistochemistry using an HA-tag-specific antibody for APPsα detection. In line with our previous work, we observed widespread expression of HA-APPsα in the dorsal hippocampus within all hippocampal subfields (CA1, CA3, and DG, see Figure 3D). Higher magnification revealed intense HA-APPsα staining in cell bodies of pyramidal (Figures 3D, E) and granule cells, while membrane-anchored Venus showed a dendritic localization. Previously, we had shown that vector derived HA-APPsα is localized to intracellular membrane compartments (ER and Golgi), consistent with the transport of APPsα within the secretory pathway to the cell surface, resulting in secretion of HA-APPsα into the extracellular space (Richter et al., 2018). Double immunostaining confirmed the neuron-specific expression of APPsα driven by the synapsin promoter, as shown by co-localization of the HA-APPsα signal with the neuronal marker NeuN (Figure 3E). Consistent with this, HA-APPsα was not detectable in microglia (Iba1, Figure 3F) nor astroglia (GFAP, Figure 3G).

Efficient expression of HA-APPsα was further confirmed by Western blot analysis (Figures 3H, I). AAV-APPsα injected THY-Tau22 mice revealed intense bands when stained with an anti-HA-tag antibody. To quantify vector mediated HA-APPsα relative to endogenous mouse APP we used the antibody M3.2 recognizing an epitope located at the 15 N-terminal residues of Aβ. This epitope is present in endogenous murine APP full length, endogenous APPsα and vector-derived APPsα (Figure 3H). Quantification revealed a 3.2-fold increase in M3.2 immunoreactivity for AAV-APPsα injected THY-Tau22 mice as compared to THY-Tau22 mice injected with AAV-Venus control vector (Figure 3I; THY-Tau22-Venus 134.65 ± 8.39% vs. THY-Tau22-APPsα 319.83 ± 46.13%, ^***^p = 0.0002). As expected, no difference in M3.2 staining was detectable between transgenic THY-Tau22 mice and wild type littermate control mice that both received AAV-Venus (Figure 3I; LM-Venus 100.00 ± 8.67% vs. THY-Tau22-Venus 134.65 ± 8.39%, *p* = 0.65, ns).

**FIGURE 4:**
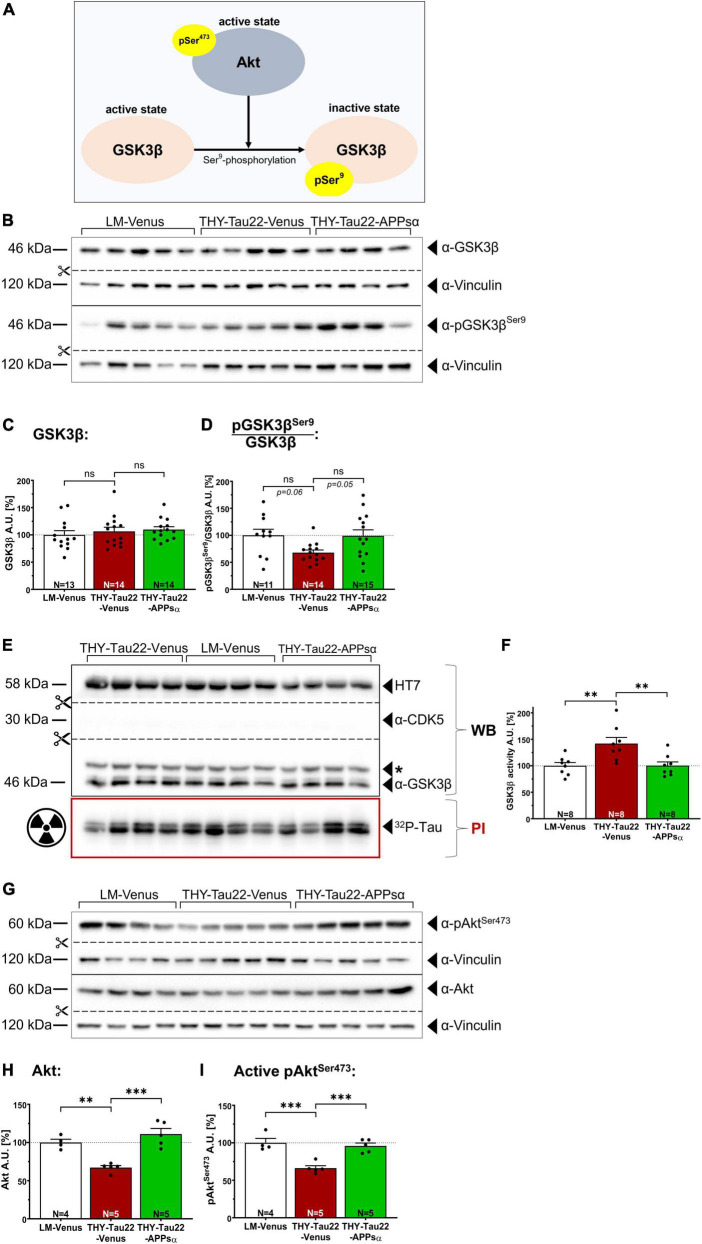
APPsα restores normal GSK3β activity and modulates the Akt/GSK3β pathway in THY-Tau22 mice. **(A)** Schematic overview of the regulation of GSK3β activity. Activated Akt (pAkt^Ser473^) negatively regulates the activity of GSK3β through phosphorylation of Ser^9^, which leads to GSK3β inactivation. **(B)** Western blot analysis of hippocampi from AAV-Venus or AAV-APPsα injected littermates (LM) or THY-Tau22 mice. Specific antibodies were used to detect total GSK3β and inactive pGSK3β^Ser9^. Vinculin is depicted as a qualitative loading control. Note that for quantification of immunoreactive bands a normalization was performed against total protein level per lane (stain-free method, Bio-Rad). **(C)** No differences were detected for total GSK3β between groups. **(D)** THY-Tau22-Venus mice revealed a strong trend toward reduced GSK3β activity, as shown by signal intensities of inactive pGSK3β^Ser9^ normalized to that of total GSK3β (LM-Venus vs. THY-Tau22-Venus, *p* = 0.060). AAV-APPsα expression restored GSK3β activity to littermate control level (THY-Tau22-Venus vs. THY-Tau22-APPsα, *p* = 0.051). **(E)** Radioactive kinase assay involving Western blot (WB) and phosphorimaging (PI) analysis after immunoprecipitation of GSK3β from AAV-Venus or AAV-APPsα injected littermates and THY-Tau22 mice. Radioactively labeled Tau was visualized using PI. Recombinant Tau (HT7), GSK3β and CDK5 were visualized by immunodetection using specific monoclonal antibodies. Note the absence of CDK5 after immunoprecipitation of GSK3β. **(F)** Quantitative analysis revealed significantly increased GSK3β activity (PI signal normalized to total immunoprecipitated GSK3β, WB signal) in THY-Tau22-Venus mice compared to LM-Venus mice (LM-Venus vs. THY-Tau22-Venus, ^**^p = 0.007). AAV-APPsα restored normal GSK3β activity (THY-Tau22-Venus vs. THY-Tau22-APPsα, ^**^p = 0.007). **(G)** Western blot analysis of total Akt and active Akt (pAkt^Ser473^) in THY-Tau22 mice after AAV-Venus or AAV-APPsα injection. Vinculin is depicted as a qualitative loading control. Note that for quantification of immunoreactive bands a normalization was performed against total protein level per lane (stain-free method, Bio-Rad). **(H,I)** Quantitative analysis of the Western blot depicted in **(G)**. THY-Tau22 mice showed a reduction in **(H)** the total expression of Akt (LM-Venus vs. THY-Tau22-Venus, ^**^p = 0.003) and **(I)** for the activating Ser^473^ phosphorylation of Akt (LM-Venus vs. THY-Tau22-Venus, ^***^p = 0.0005). AAV-APPsα rescued both total Akt and pAkt^473^ (THY-Tau22-Venus vs. THY-Tau22-APPsα, ^***^p = 0.0002 and ^***^p = 0.0009), respectively. Data are depicted as mean ± SEM; N, number of animals; age of analysis, 12 months, data were analyzed using one-way ANOVA with Tukey *post hoc* test.

Next, we asked whether APPsα may modulate GSK3β signaling in THY-Tau22 mice. First, we used Western blot analysis to assess the activation state of GSK3β (for schematic overview see Figure 4A) in hippocampal lysates of AAV-injected animals (age of injection: 9 months; age of analysis: 12 months). We found no significant difference in the total abundance of GSK3β between groups (Figure 4C; LM-Venus: 100.00 ± 7.79% vs. THY-Tau22-Venus: 106.47 ± 7.65%, *p* = 0.79, ns; and THY-Tau22-Venus 106.47 ± 7.65% vs. THY-Tau22-APPsα 109.80 ± 5.18, *p* = 0.94, ns). We then went on to measure the relative abundance of inactive GSK3β phosphorylated at Ser^9^ (pGSK3β^Ser9^) using a monoclonal antibody specifically recognizing this epitope (Figures 4B, D). THY-Tau22 mice injected with AAA-Venus showed a trend toward less inhibitory pGSK3β^Ser9^ (normalized to total GSK3β), indicating increased GSK3β activity compared to wild type littermate controls (Figure 4D; LM-Venus: 100.00 ± 11.25% vs. THY-Tau22-Venus: 67.86 ± 4.92%, *p* = 0.060, ns). AAV-APPsα led to a trend toward restored inhibitory Ser^9^ phosphorylation in THY-Tau22 mice to control level (Figure 4D, THY-Tau22-Venus: 67.86 ± 4.92% vs. THY-Tau22-APPsα 99.02 ± 11.20%, *p* = 0.05, ns). Although statistical analysis indicated that these effects did not reach significance, we observed a strong trend toward reduced GSK3β activity in transgenic THY-Tau22 mice (*p* = 0.06) and a strong trend toward a rescue by APPsα expression (*p* = 0.05). Of note, our data are also consistent with earlier studies by Ahmed et al. (2015) who found in THY-Tau22 mice enhanced hippocampal levels of activated GSK3β, as indicated by an increase in phosphorylation at the Tyr^216^ epitope and similar to our study a slight trend toward decreased phosphorylation at the inhibitory Ser^9^ epitope. Next, to further confirm and corroborate our results obtained by Western blot analysis (see Figures 4A–D) we turned again to the more sensitive *ex vivo* radioactive kinase assay to measure GSK3β activity (Figures 4E, F). As before, viral vectors were applied at 9 months and mice were sacrificed 3 months post injection. GSK3β was immunoprecipitated and its activity toward recTau was assessed by phosphorimaging and WB (see also Figure 2A for scheme). Transgenic THY-Tau22 mice that had been injected with AAV-Venus showed significantly increased GSK3β activity in this assay (LM-Venus 100.00 ± 6.14% vs. THY-Tau22-Venus 141.94 ± 11.60%, ^**^p = 0.007), consistent with increased GSK3β activity in non-injected THY-Tau22 mice (see Figures 2C, E). Strikingly, this increase in GSK3β activity was fully rescued by APPsα expression in THY-Tau22 mice, indicating its potential to normalize aberrant GSK3β activity *in vivo* (Figure 4F; THY-Tau22-Venus 141.94 ± 11.60% vs. THY-Tau22-APPsα 100.30 ± 7.07%, ^**^p = 0.007).

Having shown that APPsα rescues GSK3β activity in THY-Tau22 mice, we next studied upstream signaling components that may regulate the activation status of GSK3β. GSK3β is subject to regulation by Akt kinase (Cross et al., 1995). In its active state, when phosphorylated at Ser^473^ (pAkt^Ser473^), Akt phosphorylates GSK3β at Ser^9^ (Hermida et al., 2017) leading to GSK3β inactivation. To study Akt expression and activity, we again used Western blot analysis of hippocampal lysates of AAV-injected mice (Figures 4G–I). As compared to AAV-Venus injected control mice THY-Tau22 mice showed a significant decrease in total Akt expression (Figures 4G, H; LM-Venus 100.00 ± 4.26% vs. THY-Tau22-Venus 67.16 ± 2.66%, ^**^p = 0.003). By contrast, AAV-mediated expression of APPsα restored Akt expression to levels not significantly different from littermate control mice (Figures 4G, H; THY-Tau22-Venus 67.16 ± 2.66% vs. THY-Tau22-APPsα 111.10 ± 7.29%, ^***^p = 0.0002; LM-Venus 100.00 ± 4.26% vs. THY-Tau22-APPsα 111.10 ± 7.29%, *p* = 0.35, ns). Next, we analyzed the abundance of the activated form of Akt phosphorylated at Ser^473^ (Figure 4A) (Alessi et al., 1997; Sarbassov et al., 2005) using a monoclonal antibody that specifically detects pAkt^Ser473^. We found that Akt was significantly less phosphorylated at Ser^473^ in THY-Tau22-Venus mice compared to littermate controls (Figures 4G, I; LM-Venus 100.00 ± 5.88% vs. THY-Tau22-Venus 66.24 ± 3.27%, ^***^p = 0.0005). Again, the AAV-mediated expression of APPsα restored pAkt^Ser473^ to a degree not different from littermate controls (Figures 4G, I; THY-Tau22-Venus 66.24 ± 3.27% vs. THY-Tau22-APPsα 95.87 ± 3.86%, ^***^p = 0.0009; LM-Venus 100.00 ± 5.88% vs. THY-Tau22-APPsα 95.87 ± 3.86%, *p* = 0.78, ns).

### 2.4. APPsα restores normal level of phosphorylated β-catenin in THY-Tau22 mice

**FIGURE 5:**
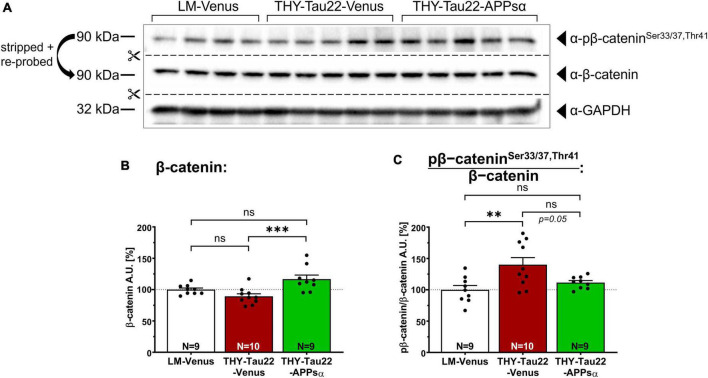
APPsα restores normal level of phosphorylated β-catenin in THY-Tau22 mice. **(A)** Western blot staining detecting the abundance of total β-catenin, or pβ-catenin^Ser33/37,Thr41^ in AAV-Venus or AAV-APPsα THY-Tau22 mice and littermate controls. GAPDH is depicted as a qualitative loading control. Note that for quantification of immunoreactive bands a normalization was performed against total protein level per lane (stain-free method, Bio-Rad). **(B)** Quantification revealed increased expression of β-catenin in THY-Tau22-APPsα mice compared to THY-Tau22-Venus mice (THY-Tau22-Venus vs. THY-Tau22-APPsα, ^***^p = 0.0009). **(C)** Quantification revealed increased pβ-catenin^Ser33/37,Thr41^ normalized to total β-catenin in THY-Tau22-Venus mice compared to LM-Venus (LM-Venus vs. THY-Tau22-Venus, ^**^p = 0.005). AAV-APPsα reduced pβ-catenin/β-catenin to levels not significantly different from that of controls (LM-Venus vs. THY-Tau22-APPsα, *p* = 0.60, ns). Data are depicted as mean ± SEM; N, number of animals; age, 12 months; data were analyzed using one-way ANOVA with Tukey *post hoc* test.

To obtain additional insight, we next asked whether APPsα expression may also modulate GSK3β activity toward other known substrates, such as β-catenin, which is involved in cell adhesion and Wnt signaling. The abundance of β-catenin is tightly regulated by phosphorylation, whereby GSK3β, in complex with Axin and APC (adenomatous-polyposis-coli), specifically phosphorylates β-catenin at Ser^33/37^ and Thr^41^, triggering its poly-ubiquitination and subsequent proteasomal degradation (Hart et al., 1998; Ikeda et al., 1998; Moon et al., 2002; Wu and He, 2006). Hence, we used Western blot analysis of hippocampal lysates of AAV-Venus or AAV-APPsα injected littermate controls or THY-Tau22 mice and used specific monoclonal antibodies to determine total β-catenin expression, as well as phosphorylated β-catenin species (pβ-catenin^Ser33/37/Thr41^; Figures 5A–C). We found no significant difference in total β-catenin between THY-Tau22 mice and littermate controls injected with AAV-Venus (Figures 5A, B, LM-Venus 100.00 ± 2.59% vs. THY-Tau22-Venus 89.33 ± 4.07%, *p* = 0.25), while AAV-APPsα slightly but significantly increased the abundance of β-catenin in THY-Tau22-mice (Figures 5A, B; THY-Tau22-Venus 89.33 ± 4.07% vs. THY-Tau22-APPsα 116.70 ± 6.57%, ^***^p = 0.0009). Consistent with increased GSK3β activity in THY-Tau22 mice, as seen in experiments with recTau as a substrate, we detected a significant increase in the ratio of pβ-catenin^Ser33/37/Thr41^/β-catenin that was reduced to littermate levels in THY-Tau22 mice upon AAV-APPsα expression (Figures 5A, C; LM-Venus 100.00 ± 6.95% vs. THY-Tau22-Venus 140.07 ± 11.33%, ^**^p = 0.0052; THY-Tau22-Venus 140.07 ± 11.33% vs. THY-Tau22-APPsα 111.55 ± 3.19%, *p* = 0.05, ns). Together our data indicate an increase in GSK3β activity in THY-Tau22 mice that can be ameliorated by APPsα.

### 2.5. APPsα rescues CDK5 hyperactivation in THY-Tau22 mice

**FIGURE 6:**
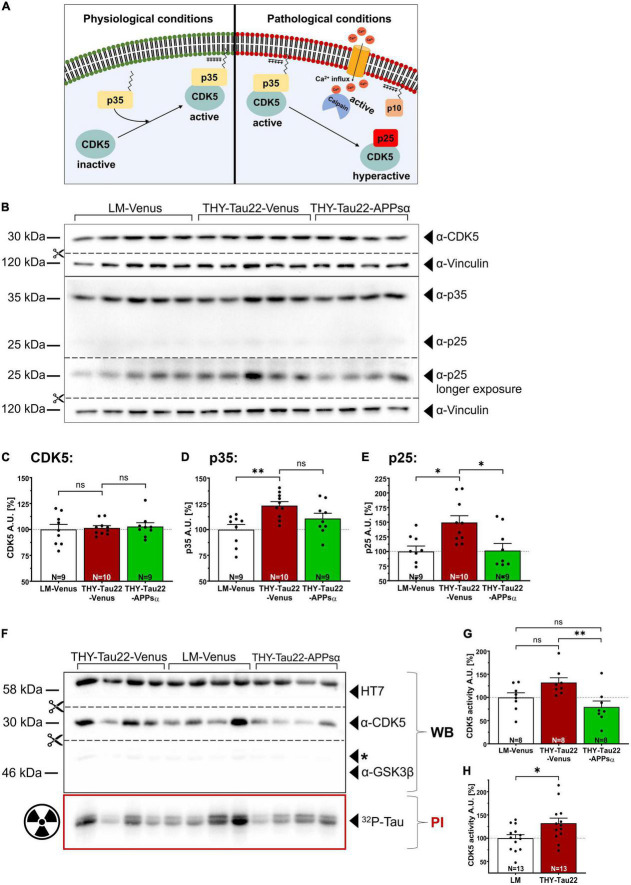
APPsα rescues CDK5 hyperactivation in THY-Tau22 mice. **(A)** Schematic overview of CDK5 regulation under physiological (left) and pathological conditions (right). Under physiological conditions, CDK5 is activated by binding to the myristoylated p35 activator, anchoring the CDK5-p35 complex to the plasma membrane. Pathological conditions lead to increased cytosolic calcium, activating the protease calpain. Active calpain cleaves p35 into p10 and p25 that remains bound to CDK5. The hyperactive CDK5-p25 complex now dissociates from the plasma membrane, shows a prolonged half-life compared to CDK5-p35 and may phosphorylate additional substrates. **(B)** Western blot analysis of hippocampal lysates of AAV-Venus or AAV-APPsα injected THY-Tau22, or littermate control mice. CDK5 and the activator proteins p35, p25 were detected using specific monoclonal antibodies. Note that a longer exposure was necessary to visualize p25 that is visible as a very faint band below p35 in the WB above. Vinculin is depicted as a qualitative loading control. Note that for quantification of immunoreactive bands a normalization was performed against total protein level per lane (stain-free method, Bio-Rad). **(C–E)** Quantitative analysis of the Western blot depicted in **(B)** revealed **(C)** a similar expression level of CDK5 between all three groups. **(D)** The abundance of p35 was significantly increased in THY-Tau22-Venus mice (LM-Venus vs. THY-Tau22-Venus, ^**^p = 0.004). **(E)** Note that THY-Tau22-Venus mice show increased expression of the hyperactivating regulatory p25 subunit that is rescued upon APPsα expression (LM-Venus vs. THY-Tau22-Venus, *p = 0.01; THY-Tau22-Venus vs. THY-Tau22-APPsα, *p = 0.01). **(F)** Radioactive kinase assay involving Western blot (WB) and phosphorimaging (PI) analysis after immunoprecipitation of CDK5 from AAV-Venus or AAV-APPsα injected littermates and THY-Tau22 mice. Radioactively labeled Tau was visualized using PI. Recombinant Tau (HT7), GSK3β and CDK5 were visualized by immunodetection using specific monoclonal antibodies. Note the absence of GSK3β after immunoprecipitation of CDK5. **(G)** Quantitative analysis revealed a slight increase in CDK5 activity (PI signal normalized to total immunoprecipitated CDK5, WB signal) in THY-Tau22-Venus mice compared to LM-Venus mice (LM-Venus vs. THY-Tau22-Venus, *p* = 0.13, ns). AAV-APPsα significantly reduced CDK5 activity to a level not different from controls (THY-Tau22-Venus vs. THY-Tau22-APPsα, ^**^p = 0.009; THY-Tau22-APPsα vs. LM-Venus, *p* = 0.414, ns). **(H)** Quantitative analysis (radioactive CDK5 kinase assay) of pooled data from non-injected and AAV-Venus injected THY-Tau22 and WT littermate mice indicates increased CDK5 activity in THY-Tau22 mice (Littermate vs. THY-Tau22, *p = 0.02). Data are depicted as mean ± SEM; N, number of animals; age, 12 months; data were analyzed using one-way ANOVA with Tukey *post hoc* test.

CDK5 is a major Tau kinase that can phosphorylate Tau at several epitopes of relevance for AD (Noble et al., 2003; Shukla et al., 2012). Conditions of stress or death signals, including exposure to Aβ lead to Ca^2+^ influx into the cell, which activates the protease calpain (Figure 6A) [(Jerónimo-Santos et al., 2015) and reviewed in Ono and Sorimachi (2012) and Pao and Tsai (2021)]. Calpain activation in turn triggers the cleavage of the CDK5 activator p35 into p25. This leads to CDK5 hyperactivation as the truncated p25 regulatory subunit increases the half-life of CDK5 and releases the hyperactive kinase from the cell membrane (Figure 6A; Patrick et al., 1999). To study whether APPsα may affect CDK5 or its neuron-specific activator proteins p35 and p25 in THY-Tau22 mice we performed Western blot analysis of hippocampal lysates from littermate control mice or THY-Tau22 mice that received viral vectors (Figures 6B–E). Expression of CDK5 was comparable between all three groups (Figures 6B, C; LM-Venus 100.00 ± 4.92% vs. THY-Tau22-Venus 101.46 ± 2.22%, *p* = 0.96, ns; THY-Tau22-Venus 101.46 ± 2.22% vs. THY-Tau22-APPsα 102.87 ± 3.65%, *p* = 0.96, ns; LM-Venus 100.00 ± 4.92% vs. THY-Tau22-APPsα 102.87 ± 3.65%, *p* = 0.85, ns). In contrast, the abundance of p35 was significantly increased in AAV-Venus injected THY-Tau22 mice as compared to littermate controls (Figures 6B, D; LM-Venus 100.00 ± 4.98% vs. THY-Tau22-Venus 123.31 ± 3.87%, ^**^p = 0.0040). An even higher increase by about 49% was observed for the truncated regulatory p25 subunit, indicating pathological hyperactivity of CDK5 in THY-Tau22 mice (Figures 6B, E; LM-Venus 100.00 ± 9.36% vs. THY-Tau22-Venus 149.49 ± 11.54%, *p = 0.011). Interestingly, THY-Tau22 mice receiving AAV-APPsα showed slightly reduced p35 abundance (Figures 6B, D; THY-Tau22-Venus 123.31 ± 3.87% vs. THY-Tau22-APPsα 110.78 ± 5.14%, *p* = 0.15, ns), while the up-regulation of p25 in THY-Tau22 mice was completely rescued to levels comparable to that of littermate control mice (Figure 6E; THY-Tau22-Venus 149.49 ± 11.54% vs. THY-Tau22-APPsα 101.58 ± 12.16%, *p = 0.014; LM-Venus 100.00 ± 9.36% vs. THY-Tau22-APPsα 101.58 ± 12.16%, *p* = 0.99, ns). Next, we studied CDK5 activity in more detail using the radioactive CDK5 pull-down assay with recTau as a substrate. Consistent with data from non-injected mice (see Figure 2) we found a slight but non-significant increase in CDK5 activity in AAV-Venus injected THY-Tau22 mice, which was prominently downregulated upon AAV-APPsα expression (Figures 6F, G; LM-Venus 100.00 ± 10.16% vs. THY-Tau22-Venus 132.15 ± 10.38%, *p* = 0.13, ns; THY-Tau22-Venus 132.15 ± 10.38% vs. THY-Tau22-APPsα 79.48 ± 12.90%, ^**^p = 0.0087, LM-Venus 100.00 ± 10.16% vs. THY-Tau22-APPsα 79.48 ± 12.90%, *p* = 0.41, ns). Finally, to increase the statistical power of the radioactive CDK5 assay, we combined the data sets from non-injected (Figure 2B) and AAV-Venus injected THY-Tau22 and WT mice (Figure 6G). This pooled data set with increased number of samples (*n* = 13) is depicted as Figure 6H and indicates that CDK5 activity is significantly increased in THY-Tau22 mice, as compared to WT littermates (LM 100.00 ± 7.52% vs. THY-Tau22 132.18 ± 10.99%, *p = 0.02). Together, our data indicate that APPsα efficiently restored normal CDK5 activity and rescued p25 hyperactivation in THY-Tau22-APPsα mice.

### 2.6. APPsα increases the abundance of soluble AT8^+^-Tau and reduces insoluble AT100^+^-Tau

**FIGURE 7:**
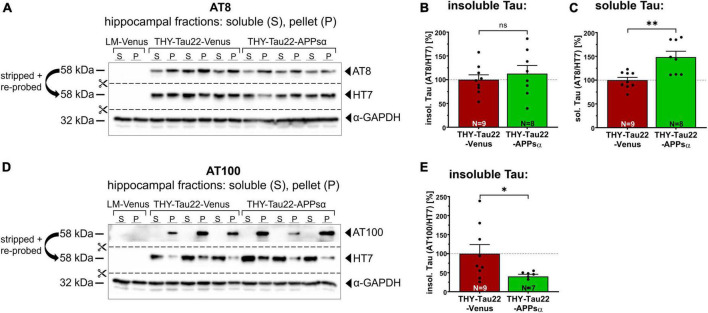
APPsα increases AT8-positive soluble Tau species and reduces AT100-positive insoluble Tau species. **(A)** Representative Western blots of hippocampal lysates from THY-Tau22 mice, injected with AAV-Venus or AAV-APPsα, probed with the AT8 antibody. After immunodetection, the membrane was stripped and re-probed with HT7 to detect total hTau. GAPDH is depicted as a qualitative loading control. Note the absence of AT8 and HT7 signal in wild type littermate controls (LM-Venus, first two lanes) indicating that antibodies specifically recognize hTau species. **(B)** AT8^+^ sarkosyl-insoluble Tau, normalized to total hTau (HT7) in the pellet fraction (P), showed no significant difference between AAV-Venus or AAV-APPsα injected THY-Tau22 mice. **(C)** Strikingly, AAV-APPsα significantly increased the amount of AT8^+^ sarkosyl-soluble Tau, normalized to total hTau in soluble fraction (S) (THY-Tau22-Venus vs. THY-Tau22-APPsα, ^**^p = 0.002). **(D)** Representative Western blots of hippocampal lysates from THY-Tau22 mice, injected with AAV-Venus or AAV-APPsα, probed with the AT100 antibody, stripped and re-probed with HT7 to detect total hTau. GAPDH is depicted as a qualitative loading control. Note the absence of AT100 and HT7 signal in LM-Venus extracts (first two lanes). No AT100-positive Tau is detected in the sarkosyl-soluble fraction despite hTau being present, indicating a high specificity for aggregated Tau species. **(E)** AAV-APPsα significantly reduced AT100^+^ sarkosyl-insoluble Tau, normalized to total hTau in the pellet fraction (THY-Tau22-Venus vs. THY-Tau22-APPsα, *p = 0.049). Data are depicted as mean ± SEM; N, number of animals; age, 12 months; data were analyzed using a two-tailed Student’s *t*-test.

Next, we asked whether the ability of APPsα to modulate kinase activity might also modulate the biochemical properties of Tau and thus the abundance of sarkosyl-soluble and sarkosyl-insoluble Tau species. Figure 7A shows a representative Western blot of hippocampal fractions probed with the AT8 antibody, stripped, and re-probed with HT7 to detect total hTau. The amount of AT8 immunoreactive insoluble Tau (normalized to the HT7 signal in the insoluble fraction) was comparable between THY-Tau22-Venus and THY-Tau22-APPsα mice (Figures 7A, B; THY-Tau22-Venus 100.00 ± 10.22% vs. THY-Tau22-APPsα 112.76 ± 17.17%, *p* = 0.52, ns). In contrast, we observed a significant increase of AT8^+^-Tau species in the sarkosyl-soluble fraction (normalized to the HT7 signal in the soluble fraction) (Figures 7A, C; THY-Tau22-Venus 100.00 ± 5.90% vs. THY-Tau22-APPsα 148.54 ± 12.33%, ^**^p = 0.002). In addition, we used the AT100 antibody to detect hTau phosphorylated at Thr^212^ and Ser^214^, AD-specific epitopes that are associated with late-stage PHF Tau and NFTs (Zheng-Fischhöfer et al., 1998; Augustinack et al., 2002). AT100-positive Tau was exclusively found in sarkosyl-insoluble fractions, consistent with its specificity of highly aggregated Tau species. Interestingly, APPsα led to a significant decrease of highly aggregated AT100^+^ insoluble Tau species (normalized to total hTau) (Figures 7D, E; THY-Tau22-Venus 100.00 ± 23.88% vs. THY-Tau22-APPsα 40.14 ± 5.72%, *p = 0.049). Together, our data indicate a change in the biochemical properties of Tau upon APPsα expression, with an increase in soluble and a decrease in insoluble Tau species.

### 2.7. APPsα reduces Tau misfolding in THY-Tau22 mice

**FIGURE 8:**
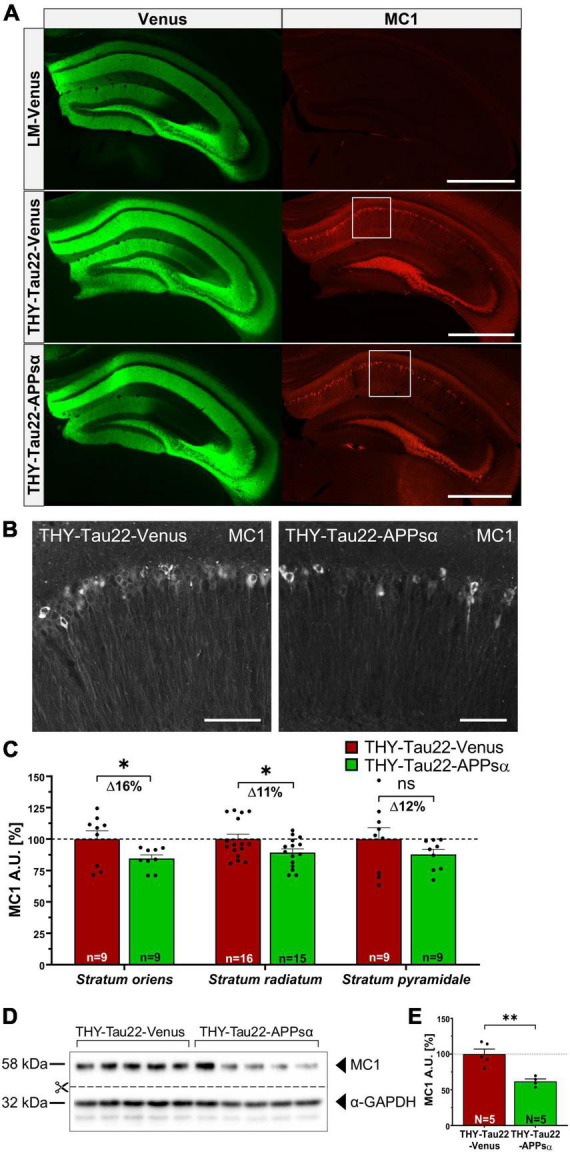
APPsα reduces Tau misfolding in THY-Tau22 mice. **(A)** Representative hippocampal brain sections from AAV-Venus injected littermate mice (top), AAV-Venus injected THY-Tau22 mice (middle), or AAV-APPsα injected THY-Tau22 mice (bottom) stained for misfolded Tau species using the MC1 antibody. AAV-Vector spread is shown by the expression of Venus. LM-Venus mice showed no specific MC1-immunoreactivity, while MC1 staining is prominently detected in THY-Tau22 hippocampus. Note that MC1 immunoreactivity is considerably reduced by APPsα. Scale bar: 500 μm. **(B)** Zoom-ins of the boxed areas indicated in **(A)** from THY-Tau22-Venus (left) and THY-Tau22-APPsα (right) mice stained with the MC1 antibody. Scale bars: 50 μm. **(C)** Quantification of mean pixel intensities obtained by MC1 staining in the stratum oriens, stratum radiatum and stratum pyramidale. Note that AAV-APPsα significant decreased MC1-immunoreactivity in the stratum oriens and stratum radiatum (THY-Tau22-Venus vs. THY-Tau-APPsα, *p = 0.048; THY-Tau22-Venus vs. THY-Tau-APPsα, *p = 0.038, respectively). Data are depicted as mean ± SEM, *n* = number of analyzed regions from 3 to 4 animals per condition, data were analyzed using a two-tailed Student’s *t*-test. **(D)** Western blot analysis of misfolded Tau species (MC1) in hippocampal lysates from THY-Tau22 mice, injected with AAV-Venus or AAV-APPsα. GAPDH is depicted as a qualitative loading control. Note that for quantification of immunoreactive bands a normalization was performed against total protein level per lane (stain-free method, Bio-Rad). **(E)** Quantitative analysis revealed a decreased abundance of MC1-positive Tau-species in THY-Tau22-APPsα mice compared to THY-Tau22-Venus mice (THY-Tau22-Venus vs. THY-Tau22-APPsα, ^**^p = 0.003). Data are depicted as mean ± SEM; N, number of animals; age, 12 months; data were analyzed using a two-tailed Student’s *t*-test.

Encouraged by the ability of APPsα to modulate Tau aggregation, we next analyzed the abundance of misfolded Tau species. We immunostained coronal brain sections of AAV-Venus or AAV-APPsα injected THY-Tau22 mice using MC1 antibody and quantified the mean fluorescence intensity of the hippocampal CA1 subfield in the stratum oriens, stratum pyramidale, and stratum radiatum (Figures 8A, B). The Venus signal confirmed the efficient transduction of neurons throughout the hippocampus using either AAV-Venus or the bicistronic AAV-APPsα vector (Figure 8A, left). Misfolded Tau species, immunodetected using the MC1 antibody (Figure 8A, right), were significantly reduced in the stratum oriens and stratum radiatum in THY-Tau22 mice that received AAV-APPsα, as compared to AAV-Venus (Figure 8C; stratum oriens: THY-Tau22-Venus 100.00 ± 6.64% vs. THY-Tau22-APPsα 84.51 ± 2.91%, *p = 0.048; stratum radiatum: THY-Tau22-Venus 100.00 ± 3.89% vs. THY-Tau22-APPsα 89.19 ± 3.00%, *p = 0.038). In stratum pyramidale, MC1^+^ Tau species were also reduced by APPsα, but values failed to reach significance (Figure 8C; THY-Tau22-Venus 100.00 ± 9.06% vs. THY-Tau22-APPsα 87.74 ± 3.98%, *p* = 0.23, ns). These results suggest that AAV-mediated expression of APPsα reduces misfolded (MC1^+^) Tau species, particularly in dendritic compartments of THY-Tau22 pyramidal neurons. In addition, AAV-APPsα also reduced the total abundance of MC1^+^ Tau species, as evidenced by Western blotting of hippocampal lysates (Figures 8D, E; THY-Tau22-Venus 100.00 ± 6.96% vs. THY-Tau22-APPsα 61.79 ± 3.43%, ^**^p = 0.0027). These data further demonstrate the potential of APPsα to reduce misfolded Tau species in THY-Tau22 mice.

### 2.8. APPsα rescues spine-density deficits and upregulates PSD95

**FIGURE 9:**
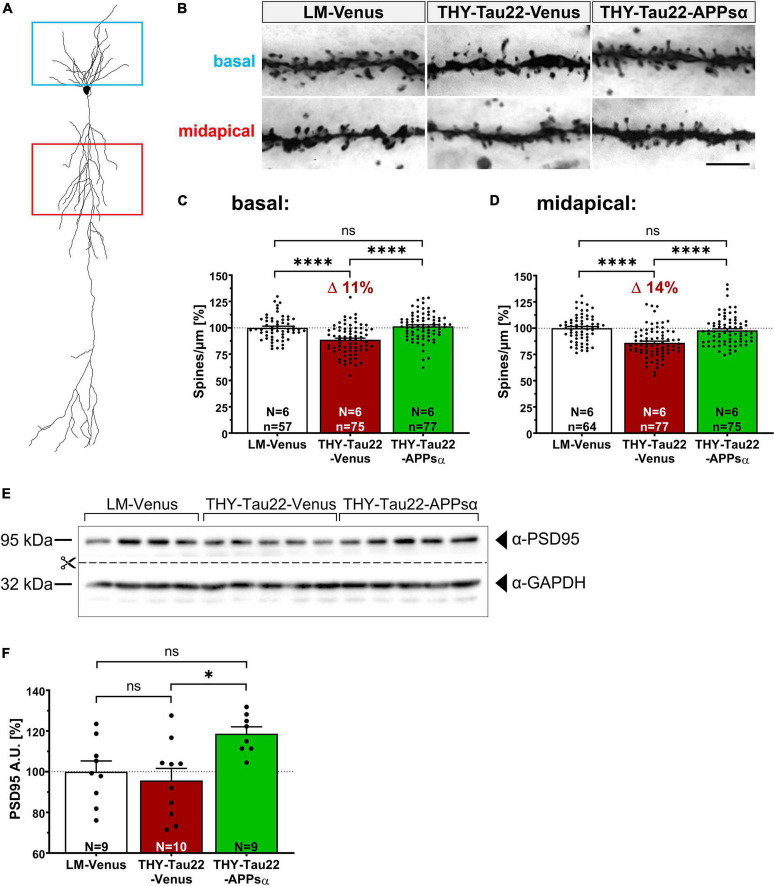
APPsα rescues spine density deficits of THY-Tau 22 mice and increases PSD95 expression. **(A)** Schematic representation of a CA1 pyramidal neuron. Boxes indicate basal (blue) and midapical (red) dendritic regions used for spine-density analysis. **(B)** Representative images of Golgi-stained basal and midapical dendritic segments of LM-Venus, THY-Tau22-Venus and THY-Tau22-APPsα mice. Images are minimum projections of z-stacks. Scale bar: 5 μm. **(C,D)** Spine-density analysis revealed a **(C)** deficit of 11% (basal) and **(D)** 14% (midapical) in THY-Tau22-Venus mice compared to LM-Venus mice (LM-Venus vs. THY-Tau22-Venus, ^*⁣*⁣**^p < 0.0001; LM-Venus vs. THY-Tau22-Venus, ^*⁣*⁣**^p < 0.0001, respectively). Both deficits were rescued by AAV-APPsα to a level not significantly different from LM-Venus mice (basal: THY-Tau22-Venus vs. THY-Tau22-APPsα, ^*⁣*⁣**^p < 0.0001; LM-Venus vs. THY-Tau22-APPsα, *p* = 0.80, ns; midapical: THY-Tau22-Venus vs. THY-Tau22-APPsα, ^*⁣*⁣**^p < 0.0001; LM-Venus vs. THY-Tau22-APPsα, *p* = 0.62, ns). **(E)** Representative Western blot analysis of PSD95 in hippocampal extracts from LM-Venus, THY-Tau22-Venus and THY-Tau22-APPsα mice. GAPDH is depicted as a qualitative loading control. Note that for quantification of immunoreactive bands a normalization was performed against total protein level per lane (stain-free method, Bio-Rad). **(F)** Quantitative analysis showed no difference in PSD95 expression between LM-Venus and THY-Tau22-Venus mice (LM-Venus vs. THY-Tau22-Venus, *p* = 0.82, ns), whereas AAV-APPsα increased the abundance of PSD95 in THY-Tau22 mice compared to AAV-Venus-injected THY-Tau22 mice (THY-Tau22-Venus vs. THY-Tau22-APPsα, *p = 0.013). Data are depicted as mean ± SEM; N, number of animals; age, 12 months; data were analyzed using one-way ANOVA with Tukey *post hoc* test.

To investigate whether the beneficial effects of APPsα are also reflected at the functional level, we studied spine density that correlates with the number of excitatory synapses. Previous studies had indicated that THY-Tau22 mice exhibit deficits in spine density starting at 9 months of age [see Figure 3A and Burlot et al. (2015)]. We used Golgi staining to analyze spine density in midapical and basal dendrites of CA1 pyramidal neurons from THY-Tau22 mice and littermates injected with either AAV-Venus or AAV-APPsα at 9 months of age and sacrificed at 12 months of age (Figures 9A, B). Consistent with previous studies, we detected a significant reduction in spine density by 11% in basal dendritic segments of THY-Tau22 mice injected with AAV-Venus as compared to AAV-Venus injected littermates (Figure 9C; LM-Venus 100.00 ± 1.51% vs. THY-Tau22-Venus 88.65 ± 1.58%, ^****^p < 0.0001). In addition, spine density in apical dendrites was reduced by about 14% in neurons of THY-Tau22 that received AAV-Venus control vector (Figure 9D; LM-Venus 100.00 ± 1.59% vs. THY-Tau22-Venus 86.06 ± 1.61%, ^****^p < 0.0001). Strikingly, AAV-mediated expression of APPsα significantly increased basal and midapical spine densities in THY-Tau22 mice to a level not significantly different from that of littermate controls (Figures 9C, D; basal: THY-Tau22-Venus 88.65 ± 1.58% vs. THY-Tau22-APPsα 101.42 ± 1.46%, ^****^p < 0.0001; LM-Venus 100.00 ± 1.51% vs. THY-Tau22-APPsα 101.42 ± 1.46%, *p* = 0.80, ns; midapical: THY-Tau22-Venus 86.06 ± 1.61% vs. THY-Tau22-APPsα 97.84 ± 1.60%, ^****^p < 0.0001; LM-Venus 100.00 ± 1.59% vs. THY-Tau22-APPsα 97.84 ± 1.60%, *p* = 0.62, ns). In addition, we analyzed by Western blot the expression of PSD95, a postsynaptic scaffolding protein that is specific for glutamatergic neurons (Hunt et al., 1996). We found that the expression of PSD95 was not altered in THY-Tau22 mice compared to littermate controls (Figures 9E, F; LM-Venus 100.00 ± 5.28% vs. THY-Tau22-Venus 95.70 ± 5.93%, *p* = 0.82, ns). However, PSD95 was significantly upregulated in THY-Tau22 mice injected with AAV-APPsα (Figures 9E, F; THY-Tau22-Venus 95.70 ± 5.93% vs. THY-Tau22-APPsα 118.69 ± 3.37%, *p = 0.012). In summary, injection of AAV-APPsα in THY-Tau22 mice efficiently rescued spine density deficits, accompanied by an increase in PSD95 expression. Thus, APPsα shows therapeutic potential to restore synapses in the presence of severe Tau pathology.

## 3. Discussion

Here, we studied the potential of neurotrophic, secreted APPsα to ameliorate aberrant kinase activity, Tau-induced pathology and synaptic deficits in THY-Tau22 mice. We followed a therapeutic strategy in which we used an AAV9 based vector to express APPsα in the hippocampus of THY-Tau22 mice at 9 months of age, a time point of established Tau pathology, and studied potential therapeutic effects 3 months later.

Our study revealed several novel findings: (1) THY-Tau22 mice exhibit increased activity of GSK3β and CDK5. (2) We show that APPsα expression modulates Akt/GSK3β signaling in THY-Tau22 mice and restores normal GSK3β activity when expressed *in vivo*, both toward recTau as a substrate and endogenous β-catenin. (3) Further, CDK5 regulatory proteins p35 and p25 were found upregulated in THY-Tau22 hippocampus, indicating CDK5 hyperactivation. Strikingly, APPsα rescued CDK5 hyperactivation in THY-Tau22 hippocampus and reduced p25 expression to levels not significantly different from wild type controls. (4) The ability of APPsα to restore normal GSK3β and CDK5 activity was paralleled by an increase in soluble AT8^+^ and a decrease in insoluble AT100^+^ Tau species, as well as a decrease in the abundance of misfolded (MC1^+^) Tau isoforms. (5) Finally, at the functional level, we show that APPsα leads to an increase in PSD95 and restores normal spine density in aged THY-Tau22 mice.

Previous studies indicated that APPsα can inhibit GSK3β activity, when applied to SH-SY5Y cells or cultured neurons *in vitro* (Deng et al., 2015). In addition, the same study also showed that overexpression of APPsα in a mouse model with Aβ plaque pathology (APP_SWE_PS1ΔE9) reduced phospho-Tau^Thr231^ in entorhinal cortex. This effect was, however, attributed to an inhibitory function of APPsα toward BACE1, that binds APPsα at an allosteric binding site leading to BACE1 inhibition, reduced Aβ production and CTFβ accumulation (Obregon et al., 2012). Thus, it remained unclear whether APPsα would also have beneficial effects in the absence of Aβ pathology and directly affect Tau-induced impairments in a model of primary tauopathy. Here, we showed protective effects of APPsα *in vivo* when administered at stages of established Tau pathology. We found that THY-Tau22 mice exhibit increased GSK3β activity at 12 months of age, that can be rescued by AAV-APPsα to WT level, as assessed by a radioactive kinase assay using recTau as a substrate. Consistent with this, when assessing the abundance of inactivating pGSK3β^Ser9^ phosphorylation we observed a strong trend toward increased activity in THY-Tau22 mice, that was restored to WT level by APPsα. We hypothesize that the radioactive kinase assay might be more sensitive, as the radioactive signal is allowed to accumulate during incubation with the substrate, while Western blot detects steady state levels of pGSK3β^Ser9^. Increased GSK3β activity in THY-Tau22 mice was further corroborated by an increase in phospho-β-catenin abundance as an endogenous GSK3β substrate. Again, AAV-mediated APPsα expression restored wild type-like phospho-β-catenin levels.

What might be the mechanism of how APPsα modulates GSK3β activity? Previous studies indicated that APPsα may upregulate Akt kinase signaling (Jimenez et al., 2011; Milosch et al., 2014) that is known to reduce GSK3β activity by inhibitory Ser^9^ phosphorylation. Indeed, THY-Tau22 mice exhibited reduced expression and activity of Akt kinase, consistent with increased inhibitory pGSK3^Ser9^ phosphorylation. Interestingly, APPsα normalized Akt kinase expression and activity, suggesting that APPsα might modulate GSK3β activity via restoring normal Akt signaling. Our findings are consistent with earlier work from Jimenez et al. (2011), who showed that in neuroblastoma N2a cells APPsα activates the Akt/GSK3β pathway in a mechanism involving insulin receptor (IR) or insulin like growth factor receptor (IGF-1R). In addition, APPsα was shown to induce Akt signaling and thereby mediate neuroprotection by binding of APPsα to cell surface APP that can function as an unconventional G-protein coupled receptor, as it harbors a binding site for G_0_ in its cytoplasmic domain (Milosch et al., 2014; Copenhaver and Kögel, 2017). Clearly, more work is needed to elucidate the mechanism underlying APPsα modulation of Akt/GSK3β signaling in THY-Tau22 mice. PI3K/Akt-mediated inactivation of GSK3β has also been reported to occur downstream of EphB2 signaling, which lead to attenuated Tau phosphorylation in Tau transgenic mice (Jiang et al., 2015).

We then went on to study potential effects of APPsα on CDK5, as another major Tau kinase implicated in AD and other tauopathies. While APPsα had no effect on the expression of the CDK5 kinase subunit, we detected a significant increase in the membrane anchored regulatory p35 subunit and most importantly a prominent, about 1.5-fold upregulation of the processed p25 subunit in THY-Tau22 hippocampus, indicating CDK5 hyperactivation upon calpain cleavage in transgenic mice. Dysregulation of CDK5 activity via p25 accumulation leads to a constitutive activation and mis-localization of CDK5-p25, redirecting it to novel substrates such as Tau or MEF2 (myocyte enhancer factor 2) which was associated with neuroinflammation, impairments in LTP (long-term potentiation) and neurodegeneration (Camins et al., 2006; Sundaram et al., 2013; Pao and Tsai, 2021). Strikingly, APPsα completely rescued p25 hyperactivation and also restored wild type-like CDK5 activity toward recTau. Our findings thus confirm and extend previous *in vitro* studies that identified downregulation of p25 in a proteomic screen in which primary neurons were treated with recAPPsα. Interestingly, this APPsα mediated downregulation of p25 was lost upon genetic deletion of Sortilin like receptor SORLA (Hartl et al., 2013).

Cleavage of p35 to p25 by calpain occurs in a Ca^2+^-dependent manner, which increased Ca^2+^ resulting from neurotoxic conditions including ischemia, overexcitation, death signals, or exposure to Aβ peptides (Lee et al., 2000; Mahaman et al., 2019). Although the mechanism of how precisely APPsα counteracts p25 activation needs further investigation, the inhibitory function of APPsα on L-Type Ca^2+^ channels (LTCC) may play an important role. As such, APPsα was recently shown to restore normal Ca^2+^ levels and normalize neuronal network functions under conditions of acute hypoxia (Hefter et al., 2016; Hefter and Draguhn, 2017). Moreover, APP was reported to bind to Ca_v_1.2 type LTCCs and to suppress voltage dependent calcium influx (Yang et al., 2009). Further, APPsα may (when present in high nanomolar to micromolar concentrations) bind to presynaptic GABA_B_R1a receptors and thereby limit excessive glutamate release (Rice et al., 2019), which may inturn result in Ca^2+^ overload and excitotoxicity in postsynaptic neurons (Fairless et al., 2014). In this regard it is noteworthy that overexpression of APPsα was shown to inhibit glutamate induced and NMDAR dependent CDK5 activation in N2a cells (Han et al., 2005). Together, multiple lines of evidence indicate that APPsα regulates Ca^2+^ homeostasis at several levels which may counteract CDK5 dysregulation. Our findings that APPsα exerts beneficial effects via normalizing both GSK3β and CDK5 activity are also highly consistent with effects observed in transgenic mice that were treated with small molecule diaminothiazole inhibitors with dual specificity for GSK3β and CDK5 (Zhang et al., 2013). Moreover, our finding of restored spine density upon APPsα mediated normalization of Tau kinase activity is further supported by work from of Ahmed et al. (2015). This study investigated a form of long-term depression (late phase L-LTD) in the hippocampus of THY-Tau22 mice and reported impairments in L-LTD in THY-Tau22 mice of similar age as studied here. Interestingly, and highly consistent with our results, impaired LTD could be rescued by acute application of a specific GSK3β inhibitor, suggesting that acute normalization of increased GSK3β activity restores synaptic plasticity. In future studies it will be interesting to see whether APPsα can not only rescue spine density, as shown here, but may also normalize LTD in THY-Tau22 mice.

With regard to AD it is interesting that Aβ was shown to activate CDK5 (Lee et al., 2000; Shukla et al., 2012) whereas this study indicates that APPsα rescues p25 hyperactivation, suggesting that an imbalance in non-amyloidogenic α-secretase processing versus β-/γ-secretase processing may lead to CDK5 dysregulation and contribute to pathological signaling cascades [see also review by Mockett et al. (2019)]. Indeed, α-secretase attenuating mutations within the prodomain of ADAM10, the major α-secretase that generates secreted APPsα, were identified in families with late onset AD (Suh et al., 2013; Agüero et al., 2020) and more recently, ADAM10 was also linked to sporadic AD through genome-wide association studies (Kunkle et al., 2019). Similarly, preclinical studies indicated that blocking the transport of ADAM10 to the cell surface and thereby inhibiting APPsα secretion, enhanced Tau phosphorylation in wild type mice (Epis et al., 2010).

A further key finding of this study was that APPsα increased the abundance of soluble AT8^+^-Tau and reduced insoluble AT100^+^-Tau. Moreover, AAV-APPsα reduced misfolded (MC1^+^) Tau species in THY-Tau22 mice, as evidenced by Western blotting and using immunohistochemistry, particularly in dendritic compartments of CA1 pyramidal neurons. This suggests that APPsα may in turn ameliorate pathological effects induced by Tau aggregation and misfolding, which likely contributes to the beneficial effects of APPsα. It will be interesting to see, whether preventive application of APPsα, before overt Tau pathology, may lead to even more pronounced effects. Finally, at the functional level, we show that AAV-APPsα increased PSD95 expression, a major postsynaptic scaffolding protein and restored normal spine density in THY-Tau22 mice. It is conceivable that normalizing p25 contributes to this effect, as conversely, inducible activation of CDK5 in the striatum was shown to reduce dendritic spine density (Meyer et al., 2008).

Due to the highly plastic nature of synapses, synaptic dysfunction and synapse loss are reversible processes even in the presence of established pathology, which is highly relevant when treating symptomatic patients. Importantly, the rescue of spine density by APPsα is not limited to THY-Tau22 mice, as we recently showed similar spine density restoring properties of APPsα also in Tau transgenic P301S mice (Bold et al., 2022). Moreover, APPsα also normalized spine density under conditions of Aβ plaque pathology (Fol et al., 2016) and in gene targeted mice lacking endogenous APP and APLP2 (Richter et al., 2018), indicating a more general function for synaptic repair. Together our findings suggest that APPsα holds therapeutic potential to mitigate aberrant kinase activation and Tau-induced synaptic pathology in primary tauopathies and AD.

## 4. Materials and methods

### 4.1. Ethics statement

All animal experiments were performed in accordance with the guidelines and regulations set forth by the German Animal Welfare Act and the Regierungspräsidium Karlsruhe (Germany) to reduce the numbers of animals and prevent unnecessary suffering. All procedures performed were approved by the Regierungspräsidium Karlsruhe (Aktenzeichen 35-9185.81/G-153/16, 35-9185.81/G-269/20).

### 4.2. Mice

Heterozygous transgenic THY-Tau22 mice (THY-Tau22) of both sexes and a corresponding number of age-matched transgene-negative littermates (LM) as a control group were used in this study. The generation and genotyping of THY-Tau22 mice were described previously (Schindowski et al., 2006). Briefly, THY-Tau22 mice overexpress the 412 amino acids human 1N4R Tau isoform, double-mutated at the residues G272V and P301S, under the Thy 1.2 promotor leading to a brain-specific expression of the transgene from postnatal day 6 onwards. All mice were genotyped using PCR on DNA obtained from ear punches (Schindowski et al., 2006). The mouse line was a kind gift from Luc Buée.

All animals had *ad libitum* access to food and water and were housed in a 12 h light/dark cycle in Makrolon Type II cages with standard bedding. Mice were used at the age of 3, 6, 9, and 12 months for the basal characterization (Tau pathology, microgliosis, and astrogliosis). Mice were injected at 9 months of age and sacrificed 3 months post-injection at 12 months of age.

### 4.3. Protein extraction based on solubility

Sarkosyl-insoluble and sarkosyl-soluble Tau aggregates were separated as described previously (Eckermann et al., 2007; Bold et al., 2022). For the Tau fractionation, mice were sacrificed by cervical dislocation 3 months post-injection at 12 months of age and the brain was rapidly removed. The cerebral hemispheres were separated, the hippocampi were dissected, snap frozen in liquid nitrogen and stored at −80°C until use. Hippocampal tissues were homogenized in 250 μl of buffer H (10 mM Tris, pH 7.4, 0.8 M NaCl, 1 mM PMSF, 10% sucrose, protease, and phosphatase inhibitors) using a bead mill (Omni Bead Ruptor 24, Omni International; 2 s × 20 s at 3.1 m/s). The homogenates were kept on ice for 20 min. Subsequently, they were centrifuged for 20 min at 21,200 × *g* at 4°C and the supernatants (S1) were collected. The resulting pellets were homogenized a second time in 250 μl of buffer H and centrifuged again. The supernatants (S2) were added to the first supernatants (S1) and transferred to a new tube. The beads were washed in 50 μl buffer H, that was added to the supernatants afterward. The combined supernatants (S1 + S2) were adjusted to 1% sarkosyl and incubated for 1 h at 37°C on an orbital shaker at 350–400 rpm. Subsequently samples underwent ultracentrifugation for 68 min at 127,900 × *g* at 4°C. Supernatants (S) contained sarkosyl-soluble Tau species and pellets (P) contained sarkosyl-soluble Tau species. The pellets (P) were resuspended in 33 μl Tris-buffered saline (10 mM Tris, 154 mM NaCl, and pH 7.4) and 30 μl were used directly for SDS-PAGE loading. From the supernatants (S), 5 μl were used for protein quantification using a BCA assay (#QPBCA-1KT, Sigma-Aldrich). The fractionation, protein quantification and sample preparation for SDS-PAGE was completed on the same day.

### 4.4. Hippocampal tissue samples used for Western blot analysis

Mice were sacrificed 3 months post-injection at 12 months of age by cervical dislocation. The hippocampus was dissected and snap frozen in liquid nitrogen until handling. Hippocampal tissue samples were prepared by homogenization in 250 μl of RIPA buffer [50 mM Tris, pH 7-8, 150 mM NaCl, 0.1% (v/v) SDS, 0.5% (v/v) dichloroacetic acid, 1% NP-40, cOmplete protease inhibitor cocktail (#04693116001, Sigma Aldrich), PhosStop (#4906845001, Sigma-Aldrich)] using a bead mill (Omni Bead Ruptor 24, Omni International; 2 s × 20 s at 3.1 m/s). Afterward, tissue and cell debris were removed by centrifugation at 5,000 *g* for 10 min at 4°C, and the supernatants were collected. Following protein quantification using a BCA assay (#QPBCA-1KT, Sigma-Aldrich), the homogenates were used for sample preparation.

### 4.5. Western blot analysis

Hippocampal tissue samples (20 μg/20 μl) or sarkosyl-soluble (S) and sarkosyl-insoluble (P) fractions (both 20 μg/20 μl) were used for SDS-PAGE. Proteins were separated using 4% (stacking gel) and 10% (running gel) Tris-glycine gels in Laemmli buffer. The running gels contained TCE (Trichlorethanol, Merck Millipore) which allowed the total protein detection after electrophoresis (1 min of activation) and blotting using an UV transilluminator (Gürtler et al., 2013) (ChemiDoc MP system, Bio-Rad, Hercules, CA, United States). Proteins were transferred onto PVDF membranes (0.45 μm, GE Healthcare) using a wet/tank electroblotting system at 450 mA for 1 h. Membranes were subsequently blocked in PBS-T (0.05% Tween-20 in PBS) with either 3% dried milk powder or 5% BSA for 1 h at room temperature (RT) and then incubated with primary antibodies, diluted in PBS-T with either 3% dried milk powder or 5% BSA at 4°C overnight. The following antibodies were used: AT8 (mouse monoclonal, 1:500, #MN1020, Thermo Fisher Scientific), AT180 (mouse monoclonal, 1:1000, #MN1040, Thermo Fisher Scientific), HT7 (mouse monoclonal, 1:1000, #MN1000, Thermo Fisher Scientific), MC1 (mouse monoclonal, 1:500, kindly provided by Peter Davies), α-CDK5 (mouse monoclonal, 1:1000, #sc-6247, Santa Cruz Biotechnology), α-p35/p25 (rabbit monoclonal, 1:1000, #2680, Cell Signaling Technology), α-Vinculin (mouse monoclonal, 1:1000, #sc-73614, Santa Cruz Biotechnology), α-GAPDH (rabbit polyclonal, 1:2000, #ABS16, Merck Millipore), α-beta-Tubulin (mouse monoclonal, 1:10000, #MAB3408, Merck Millipore), α-GSK3β (mouse monoclonal, 1:1500, #9832, Cell Signaling Technology), α-pGSK3β^Ser9^ (rabbit monoclonal, 1:1000, #9322, Cell Signaling Technology), α-GFAP (rabbit polyclonal, 1:1000, #173002, Synaptic Systems), α-HA-tag (mouse monoclonal, 1:1000, #2367, Cell Signaling Technology), α-PSD95 (mouse monoclonal, 1:1000, #MAB1598, Merck Millipore), α-β-catenin (mouse monoclonal, 1:500, #sc-7963, Santa Cruz Biotechnology), α-pβ-catenin^Ser33/37/Thr41^ (rabbit polyclonal, 1:1000, #9561, Cell Signaling Technology), M3.2 (mouse monoclonal, 1:1000, a kind gift from Paul Mathews), α-Akt (rabbit monoclonal, 1:1500, #4691, Cell Signaling Technology), α-pAkt^Ser473^ (rabbit monoclonal, 1:1000, #4060, Cell Signaling Technology). On the next day, membranes were washed with PBS-T, incubated with horseradish peroxidase-coupled (HRP-coupled) secondary antibodies (goat-α-mouse HRP, 1:10000, #1151-165-146, Dianova; donkey-α-rabbit HRP, 1:10000, #711-035-152, Dianova), washed again and developed using SignalFire ECL reagent (#6883, Cell Signaling Technology), or SignalFire Plus ECL reagent (#12630, Cell Signaling Technology). Signals were detected using the Bio-Rad Chemidoc MP imager (Bio-Rad, Hercules, CA, United States).

Prior to re-probing the membranes were incubated in stripping buffer [62.5 mM Tris, pH 6.7, 2% (w/v) SDS, 100 mM β-mercaptoethanol) for 30 min at 65°C. After washing with PBS-T for 30 min, the membranes were blocked again in PBS-T with 3% dried milk powder for 1 h at RT and incubated with primary antibodies diluted in blocking buffer overnight at 4°C. On the next day, membranes were washed, incubated with secondary antibody and imaged as described above.

### 4.6. Radioactive kinase activity assay

The kinase activity assay was modified from Bankston et al. (2017). First, hippocampal tissue was homogenized in 300 μl of lysis buffer [0.05 M Tris-HCl, pH 7.5, 0.25 M NaCl, 10 mM EDTA, 5 mM Na_3_VO_4_, 5 mM NaF, 0.1% (v/v) NP-40, 1 mM PMSF, 1 μg/μl Pepstatin/Leupeptin/Aprotinin] using a bead mill (Omni Bead Ruptor 24, Omni International; 2 s × 20 s at 3.1 m/s). The lysates were transferred to a fresh tube and the beads were washed in 50 μl of lysis buffer, that was added to the corresponding lysates afterward. Following a centrifugation step at 10,000 × *g* for 10 min at 4°C, the supernatants were transferred to fresh tube. Subsequently a pre-clearing step was performed to avoid unspecific binding of proteins to the beads. For this supernatans were incubated with 50 μl of a 50% slurry of Protein A beads [Protein-A-Sepharose CL-4B, GE17-0780-01, Merck Millipore, used for immunoprecipitation (IP) of GSK3β], or 50 μl of a 50% slurry of Protein G beads [Protein-G-Sepharose 4 Fast Flow, GE17-0618-01, Merck Millipore, used for IP of CDK5). The mixtures were rotated at 4°C for 30 min and subsequently centrifuged at 3,000 × *g* for 2 min at 4°C. The supernatants were transferred to a fresh tube and a BCA assay (#QPBCA-1KT, Sigma-Aldrich) was used to quantify the concentration of protein. 650 μg of total protein from the lysates in equal volume of lysis buffer (500 μl) was used for immunoprecipitation of CDK5 or GSK3β. The lysate of one hippocampus was used for two rounds of immunoprecipitation. As samples often contained insufficient protein concentrations for two rounds, only the first round was performed with equal protein amounts, while the remaining protein was used for the second round, resulting in different protein input and amount of immunoprecipitated kinase. To account for this input variability a normalization was performed during quantitative data analysis (see 4.11.2 Radioactive kinase activity assay). For the immunoprecipitation, 2 μg of primary antibody (α-CDK5, #sc-6247, Santa Cruz Biotechnology; α-GSK3β, #9832, Cell Signaling Technology) were added to each sample. The mixtures were rotated for 3 h at 4°C, before 50 μl of a 50% slurry of Protein A/G beads was added. The mixtures were again rotated for 2 h at 4°C and centrifuged at 3,000 × *g* for 2 min at 4°C. The supernatants were discarded, and the beads were washed three times using 500 μl of lysis buffer (rotated for 10 min in each washing step). The tubes were centrifuged at 3,000 × *g* for 2 min at 4°C and the supernatants were discarded. Afterward, the beads were equilibrated to kinase buffer via washing the beads twice in 500 μl of kinase buffer [0.02 M MOPS, 5 mM MgCl_2_, 0.1 mM EDTA, 0.1 mM EGTA, 0.01% (v/v) PMSF] without ATP or recombinant Tau for 10 min each at 4°C and centrifuged again. Then the beads were resuspended in 25 μl kinase buffer containing 3 μg/25 μl recombinant Tau (#SP-501-100, Boston Biochem), 25 μM ATP (#PV3227, Thermo Fisher Scientific) and 0.5 μCi of [γ-^32^P]ATP. The reaction was incubated at 30°C for 30 min. To stop the reaction, 5 μl of 5× Laemmli buffer [0.5 M Tris-HCl, 17% (v/v) glycerol, 10% (w/v) SDS, 0.05% (v/v) bromophenol blue, 42% (v/v) β-mercaptoethanol] was added to each reaction, and the samples were boiled for 10 min at 95°C. All reactions were immediately resolved by 12% SDS-PAGE and transferred to 0.45 μM PVDF-Membrane as described above. The membranes were exposed to phosphor screen at 20°C and scanned using a phosphorimager (Typhoon FLA 9500, GE Healthcare). After phosphorimaging, Western blot analysis was used to assess immunoprecipitation as described above. For this assay the following primary antibodies were used: α-CDK5 (mouse monoclonal, 1:1000, #sc-6247, Santa Cruz Biotechnology), α-GSK3β (mouse monoclonal, 1:1500, #9832, Cell Signaling Technology), HT7 (mouse monoclonal, 1:1000, #MN1000, Thermo Fisher Scientific). For the densitometric analysis the Bio-Rad Image Lab software was used (Version 6.1.0, build 7, standard edition).

### 4.7. Immunohistochemistry (IHC)

Non-injected littermates and THY-Tau22 mice of 3, 6, 9, and 12 months of age were sacrificed on the same day and brain sections were stained in parallel. Stereotactically injected mice for IHC were sacrificed at 12 months of age. For IHC, mice were sacrificed via carbon dioxide inhalation and immediately transcardially perfused with ice-cold PBS, followed by 4% PFA in PBS. Brains were dissected and post-fixated in 4% PFA in PBS for 24 h at 4°C. 40 μm coronal brain sections were cut using a vibratome (HM650V Vibratome, Thermo Fisher Scientific) and collected in PBS with 0.05% NaN_3_. Sections were stained free-floating in 24-well plates.

#### 4.7.1. Standard IHC

For standard IHC (staining of IBA1, GFAP, HA-tag, and NeuN) sections were incubated in blocking/permeabilization buffer (5% BSA, 5% NGS, and 0.4% Triton X-100 in PBS) for 2 h at RT. Afterward, sections were incubated with primary antibodies in PBS with 5% NGS and 0.2% Triton X-100 overnight at 4°C on a shaker. The following antibodies were used: α-IBA1 (rabbit polyclonal, 1:1000, #234003, Synaptic Systems), α-GFAP (mouse monoclonal, 1:1000, #G3893, Sigma-Aldrich), α-HA-tag (rabbit monoclonal, 1:500, #3724, Cell Signaling Technology), α-NeuN (mouse monoclonal, 1:1500, #MAB377, Merck Millipore). After washing in PBS, the sections were incubated with corresponding secondary antibodies, diluted in PBS with 0.1% BSA and 0.05% Triton X-100 for 2 h at RT. The following secondary antibodies were used: α-mouse-Cy5 (goat α-mouse IgG, Cyanine5 coupled, 1:1000, #A10524, Thermo Fisher Scientific), α-rabbit-Alexa568 (goat α-rabbit IgG, Alexa Fluor 568 coupled, 1:1000, #A11011, Thermo Fisher Scientific), α-rabbit-Cy3 (goat α-rabbit IgG, Cyanine3 coupled, 1:1000, #A711-165-152, Jackson ImmunoResearch). Sections were washed and nuclei were counterstained with DAPI (diluted 1:5000 in PBS, 10 min at RT). After another washing step, sections were mounted in Mowiol on Superfrost microscope slides (Menzel). Images were taken with an Axio Observer Z1 microscope (Zeiss, Germany) controlled using the software AxioVision (Release 4.8.2 SP3, 32-bit).

#### 4.7.2. Fluorescent ABC staining

For all Tau-stainings a fluorescent Avidin-Biotin-Complex Kit (ABC Kit, Vectastain, #PK-4000, Vector Laboratories) was used and all steps were performed at RT. Sections were incubated in 50 mM NH_4_CL for 15 min, neutralizing remaining PFA, before they were washed in PBS and permeabilized (2% Triton X-100 in PBS) overnight, followed by a blocking step [M.O.M. (Mouse on Mouse) Blocking Reagent, 1:100, #MKB-2213, Vector Laboratories] for 6 h. After washing, sections were incubated with the following primary antibodies in PBS with 5% NGS: HT7 (mouse monoclonal, 1:200, #MN1000, Thermo Fisher Scientific), AT8 (mouse monoclonal, 1:200, #MN1020, Thermo Fisher Scientific), AT180 (mouse monoclonal, 1:200, #MN1040, Thermo Fisher Scientific), MC1 (mouse monoclonal, 1:50, kindly provided by Peter Davies). On the next day, sections were washed again in PBS and then incubated in biotinylated secondary antibody (1:250, goat α-mouse IgG antibody, biotinylated, #BA-9200, Vector Laboratories) for 2 h (3% NGS and 1% BSA in PBS). Afterward, the sections were washed again and incubated in ABC solution (1:100, Vectastain ABC Kit, #PK-4000, Vector Laboratories) for 2 h. The ABC solution was allowed to stand for 15–30 min before use. Following another washing step in PBS, a streptavidin rhodamine-RedX conjugate was used to detect the biotinylated secondary antibody (2 h in the dark, 8 μg/μl, S6366, Thermo Fisher Scientific). Afterward, sections were washed in PBS, nuclei were counterstained with DAPI (1:5000 in PBS, 10 min), washed again and mounted in Mowiol on Superfrost microscope slides (Menzel). Images were taken with an Axio Observer Z1 microscope (Zeiss, Germany) controlled using the software AxioVision (Release 4.8.2 SP3, 32-bit). The quantification of the images is described below.

### 4.8. AAV plasmid design and vector production

The murine APPsα coding sequence was codon optimized and cloned under control of the synapsin promotor into a single-stranded rAAV2-based shuttle vector, as described previously (Fol et al., 2016). Briefly, a bicistronic construct was used, harboring a T2A site that connects the cDNA of muAPPsα and lckVenus. A double HA-tag was inserted N-terminally of APPsα, enabling an easy immunodetection. A lymphocyte-specific protein tyrosine kinase (lck) peptide motif was fused to the cDNA of Venus, inducing farnesylation and subsequent membrane anchoring of the yellow fluorescent protein Venus. A monocistronic AAV-Venus vector, only encoding lckVenus, was used as a control vector. Both constructs were packed into AAV9 capsids, as previously described (Richter et al., 2018). Briefly, HEK-293 cells were transiently co-transfected with the transfer vector and the helper plasmid pDP9rs. Supernatants and cell lysates were collected 3 days post-transfection and virions were purified by ultracentrifugation on an iodixanol density gradient followed by buffer exchange to 0.01% pluronic/PBS via a 100 kDa Amicon centrifugal filter unit (Merck Millipore). Free inverted terminal repeat (ITR)-specific quantitative TaqMan PCR was used to determine the final concentration, expressed as genomic copies per μl of concentrated stocks (gc/μl) as previously described (D’Costa et al., 2016).

### 4.9. Stereotactic injection of AAVs

Under anesthesia AAV-Venus (titer 5 × 10^8^ gc/μl) or AAV-APPsα (1 × 10^9^ gc/μl) were bilaterally injected into the hippocampus at two injection spots per hemisphere, using 1 μl vector stock per spot at a rate of 0.2 μl/min. Efflux of viral vector preparations was prevented by letting the cannula rest for 1 min after completing an injection. Stereotactic coordinates of injection sites are as follows (relative to bregma): anteroposterior (A/P) −2 mm, mediolateral (M/L) ±1 mm, dorsoventral (D/V) −2.25 mm (first spot), and −1.75 mm (second spot).

### 4.10. Golgi staining

Golgi staining was done using the Rapid Golgi Staining Kit according to the manufacturer’s protocol (FD NeuroTechnologies) and as described previously (Steubler et al., 2021). Briefly, mice were sacrificed 3 months post injection at the age of 12 months. The brain was removed from the skull and one hemisphere of each mouse was used for Western blot analysis and the other hemisphere was used for Golgi staining. All procedures of the Golgi staining were performed in the dark. Impregnation solution was prepared 3 h in advance by mixing equal volumes of kit Solutions A and B and the tissue was immersed in 2.5 ml of fresh impregnation solution and incubated for 2 weeks at RT in total. After 24 h the impregnation solution was replaced. Afterward, the hemispheres were transferred into kit Solution C and stored at RT for 3 days. Solution C was replaced after 24 h. Then the hemispheres were snap-frozen on dry ice, and 100 μm coronal sections were cut using a cryotome (HM550, Thermo Fisher Scientific). Sections were mounted with Solution C on adhesive microscope slides pro-coated with 0.5% gelatin/0.05% Chromalaun and let dry at RT. The staining was performed according to the manufacturer’s protocol. Finally, sections were cleared using RotiClear (Roth) and coverslipped with Permount (Thermo Fisher Scientific).

#### 4.10.1. Image acquisition and spine density analysis

Z-stack images of hippocampal CA1 pyramidal neurons were acquired with an Axio Observer Z1 microscope (Zeiss, Germany) using a Plan Apo 63×/1.4 Oil DICII objective (Zeiss) and a z-stack thickness of 130 nm. Basal and midapical dendrites were imaged, whereby dendrites close to the soma or close to branching points (10 μm) were excluded. The exposure time was individually set for each image so that the complete range of the greyscale was used. The spine density was determined per micrometer of dendritic length using Neurolucida software (Neurolucida, Version 2019.1.2; Neurolucida Explorer, Version 2019.2.1; MicroBrightField Bioscience) as described (Steubler et al., 2021). Data acquisition and analysis were performed blind to genotype and injected viral vector.

### 4.11. Data analysis and statistics

All analyses were performed with the experimenter blinded to the genotype and experimental condition. For each experiment, the number of animals (*N*) are given in the corresponding figure. Statistical analysis was performed using GraphPad Prism (Version 8.4.3).

#### 4.11.1. Western blot experiments

For the quantification of Western blots, the Bio-Rad Image Lab software was used (Version 6.1.0, build 7, standard edition). Quantification was performed against total protein levels per lane, detected using the stain-free method (Bio-Rad), as described (Gürtler et al., 2013). In addition, immunostaining for typical housekeeping proteins (GAPDH, Vinculin, and β-Tubulin) was shown as a qualitative loading control. For the Tau sarkosyl-extraction the signal intensity of the AT8/AT100 immunoreactivity was normalized to the corresponding HT7 signal in the same fraction. GraphPad Prism (Version 8.4.3) was used to identify outliers (ROUT method, *Q* = 5%) and outliers were removed from the dataset. Data were analyzed by either a two-tailed Student’s *t*-test or by using one-way ANOVA with Tukey *post hoc* test. *p*-values of *p* ≤ 0.05 were considered significant and plotted as follows: *p ≤ 0.05; ^**^p ≤ 0.01, ^***^p ≤ 0.001, ^****^p ≤ 0.0001. All data are indicated as mean ± SEM.

#### 4.11.2. Radioactive kinase activity assay

For the quantification of kinase activity assays, the densities of autoradiographic images and immunoblots were analyzed using the Bio-Rad Image Lab software (Version 6.1.0, build 7, standard edition). To account for input variability and potential differences in pulldown efficiency signal intensity of ^32^P-recTau (PI) was normalized to total amount of immunoprecipitated kinase (WB signal intensity). GraphPad Prism (Version 8.4.3) was used to identify outliers (ROUT method, *Q* = 5%) and outliers were removed from the dataset. Data were analyzed by either a two-tailed Student’s *t*-test or by using one-way ANOVA with Tukey *post hoc* test. *p*-values of *p* ≤ 0.05 were considered significant and plotted as follows: *p ≤ 0.05; ^**^p ≤ 0.01, ^***^p ≤ 0.001, ^****^p ≤ 0.0001. All data are indicated as mean ± SEM.

#### 4.11.3. Tau immunohistochemistry

For quantification of Tau immunostainings, *z*-stack mosaic images of the hippocampus were acquired using a 10× objective (Zeiss) and consistent settings (number of steps: 11, step size: 2.7 μm) and exposure times, avoiding overexposure. Quantification was performed on maximum intensity projections of three hippocampal subregions: stratum oriens (O), stratum pyramidale (P), and stratum radiatum (R). In each of these regions, three non-overlapping rectangles were positioned, and the mean intensity was measured using Fiji (ImageJ Version 1.53t). GraphPad Prism (Version 8.4.3) was used to analyze data for Gaussian distribution using the D’Agostino-Pearson omnibus test. Outliers were identified using the ROUT method (*Q* = 5%) and outliers were removed from the dataset. Background staining was identified as immunoreactivity obtained in WT sections which was set as 100%. Staining intensity in the CA1 layers was quantified relative to WT background. For the first time point (3 months of age) significant differences were indicated relative to the WT control. For subsequent time points (6, 9, and 12 months of age) differences were calculated relative to the proceeding time point to asses age-dependent differences. Datasets were analyzed using one-way ANOVA, followed by Tukey *post hoc* test. Non-Gaussian distributed data were analyzed using the non-parametric Kruskal–Wallis test, followed by Dunn’s test for multiple comparisons. *p*-values of *p* ≤ 0.05 were considered significant and plotted as follows: *p ≤ 0.05; ^**^p ≤ 0.01, ^***^p ≤ 0.001, ^****^p ≤ 0.0001. All data are indicated as mean ± SEM. Image analysis was performed on raw data. Images depicted in the figures were subject to post-processing to enhance visibility via enhancing contrast and brightness. Figures were created using the graphics program Affinity Designer (version 1.8.6).

#### 4.11.4. Spine density analysis

Data were analyzed for Gaussian distribution using the D’Agostino-Pearson omnibus test using GraphPad Prism (Version 8.4.3) and outliers (ROUT method, *Q* = 5%) were removed. Datasets were analyzed using one-way ANOVA, followed by Tukey *post hoc* test for multiple comparisons. *p*-values of *p* ≤ 0.05 were considered significant and plotted as follows: *p ≤ 0.05; ^**^p ≤ 0.01, ^***^p ≤ 0.001, ^****^p ≤ 0.0001. All data are indicated as mean ± SEM.

## Data availability statement

The original contributions presented in this study are included in this article/Supplementary material, further inquiries can be directed to the corresponding author.

## Ethics statement

This animal study was reviewed and approved by Regierungspräsidium Karlsruhe, Referat 35, Schlossplatz 1-3, 76131 Karlsruhe, Germany.

## Author contributions

DB, CSB, LR, and JF performed the research. DB, CSB, LR, MB, JF, JJ, and SL analyzed the data. DB wrote the first draft of the manuscript. CJB and MK edited the manuscript. CJB contributed reagents and analytic tools. UM designed the research and wrote the manuscript. All authors contributed to the article and approved the submitted version.

## Supplementary material

Supplementary Figure 1 No specific anti-hTau staining was detected in littermate control mice. Brain sections from 12 months old littermate control mice were stained using distinct anti-hTau antibodies. No specific staining was observed with any of the used antibodies (HT7, MC1, AT8, and AT180). The mean fluorescence intensities (MFI) were quantified in three hippocampal layers (stratum oriens, stratum pyramidale, and stratum radiatum, see Figure 1A, left) and set to 100% for the normalization of the immunoreactivity of stained THY-Tau22 brain sections shown in Figure 1. Images were captured with the same laser intensity. Scale bars: 500 μm.
