# Supplementary figures and images for: APPsα rescues CDK5 and GSK3β dysregulation and restores normal spine density in Tau transgenic mice
Source: Front Cell Neurosci. 2023 Jan 26;17:1106176. doi: 10.3389/fncel.2023.1106176 (PMC9909437; doi:10.3389/fncel.2023.1106176)

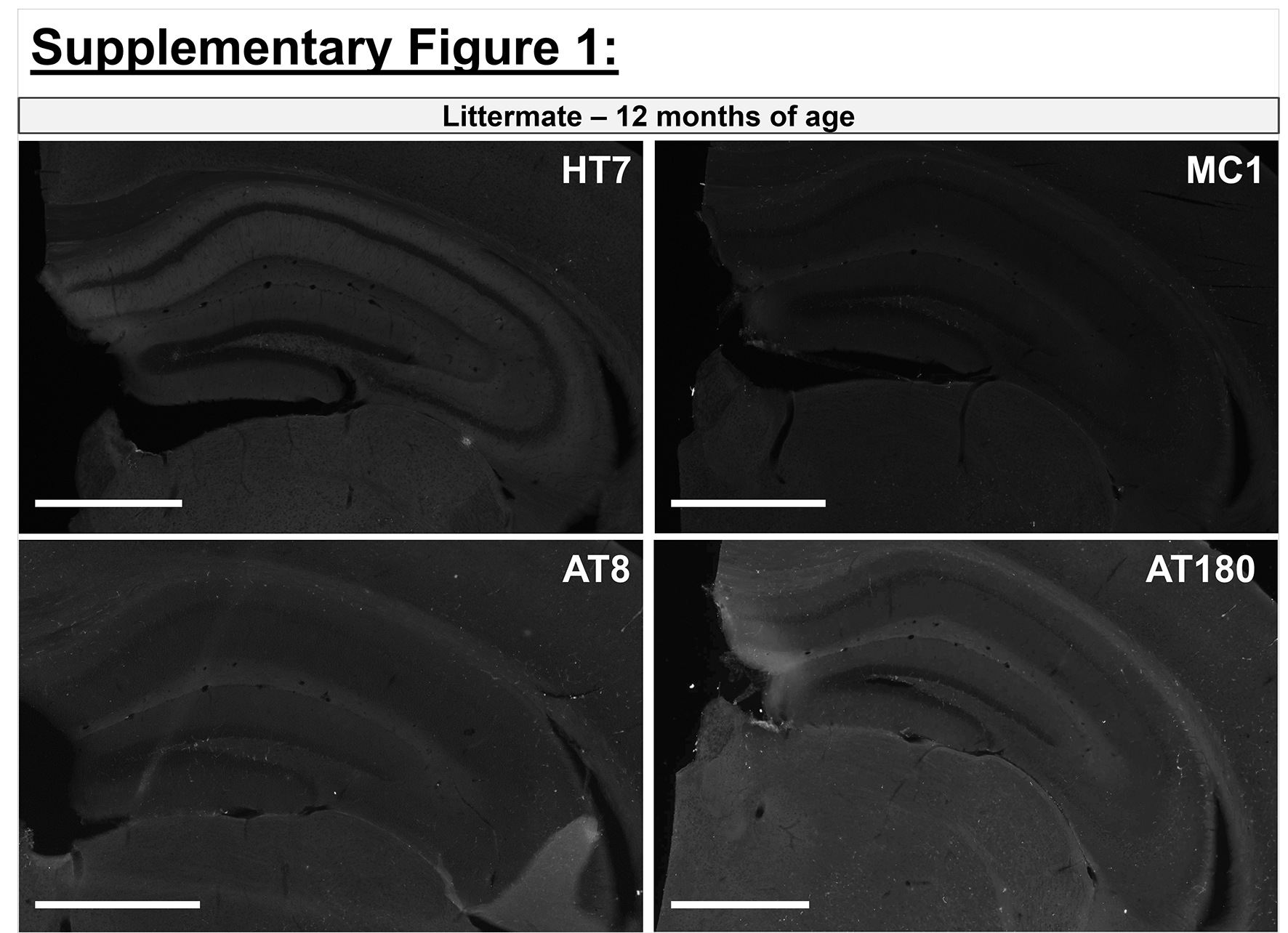

Supplement: Supplementary Figure 1 — No specific anti-hTau staining was detected in littermate control mice. Brain sections from 12 months old littermate control mice were stained using distinct anti-hTau antibodies. No specific staining was observed with any of the used antibodies (HT7, MC1, AT8, and AT180). The mean fluorescence intensities (MFI) were quantified in three hippocampal layers (stratum oriens, stratum pyramidale, and stratum radiatum, see Figure 1A, left) and set to 100% for the normalization of the immunoreactivity of stained THY-Tau22 brain sections shown in Figure 1. Images were captured with the same laser intensity. Scale bars: 500 μm. [file Image_1.tif]
